# Topics and trends in fresh tea (*Camellia sinensis*) leaf research: A comprehensive bibliometric study

**DOI:** 10.3389/fpls.2023.1092511

**Published:** 2023-04-06

**Authors:** YiQin Chen, YunFei Li, ChengWen Shen, LiZheng Xiao

**Affiliations:** ^1^ Key Laboratory of Tea Science of Ministry of Education, College of Horticulture, Hunan Agricultural University, Changsha, China; ^2^ National Research Center of Engineering and Technology for Utilization of Botanical Functional Ingredients, Changsha, China; ^3^ Co-Innovation Center of Education Ministry for Utilization of Botanical Functional Ingredients, Changsha, China; ^4^ Key Laboratory for Evaluation and Utilization of Gene Resources of Horticultural Crops, Ministry of Agriculture and Rural Affairs of China, Hunan Agricultural University, Changsha, China

**Keywords:** *Camellia sinensis*, fresh tea leaves, bibliometrics, theanine, catechins, tea quality, tea plant stress response

## Abstract

Tea plant (*Camellia sinensis*) is a widely cultivated cash crop and tea is a favorite functional food in the world. Fresh tea leaves (FTLs) play a critical role in bridging the two fields closely related to tea cultivation and tea processing, those are, tea plant biology and tea biochemistry. To provide a comprehensive overview of the development stages, authorship collaboration, research topics, and hotspots and their temporal evolution trends in the field of FTLs research, we conducted a bibliometric analysis, based on 971 publications on FTLs-related research published during 2001-2021 from Web of Science Core Collection. CiteSpace, R package Bibliometrix, and VOSviewer were employed in this research. The results revealed that the development history can be roughly divided into three stages, namely initial stage, slow development stage and rapid development stage. *Journal of Agricultural & Food Chemistry* published most articles in this field, while *Frontiers in Plant Science* held the highest total citations and h-index. The most influential country, institution, and author in this field was identified as China, the Chinese Academy of Agricultural Sciences, and Xiaochun Wan, respectively. FTLs-related research can be categorized into three main topics: the regulation mechanism of key genes, the metabolism and features of essential compounds, and tea plants’ growth and stress responses. The most concerning hotspots are the application of advanced technologies, essential metabolites, leaf color variants, and effective cultivation treatments. There has been a shift from basic biochemical and enzymatic studies to studies of molecular mechanisms that depend on multi-omics technologies. We also discussed the future development in this field. This study provides a comprehensive summary of the research field, making it easier for researchers to be informed about its development history, status, and trends.

## 1 Introduction

Tea is a commonly consumed beverage globally and is processed from the tender shoots (known as fresh leaves, usually containing a bud and one or more leaves depending on the level) of the tea plant (*Camellia sinensis*). Tea is popular with consumers for its flavor, fragrance, and bioactive compounds. Fresh tea leaves (FTLs) are used for tea processing and are rich in secondary metabolites, such as theanine, catechins, and caffeine (Li et al., 2015b). These components contribute to the distinct sensory quality and physiological functions (Zhang et al., 2019) of the tea products. Theanine is associated with the umami taste and formation of volatile compounds (Guo et al., 2018, Guo et al., 2019), while also functioning in anti-obesity and reducing stress-related symptoms (Sharma et al., 2018; Hidese et al., 2019). Catechins increase the astringent taste of tea, and oligomerization products such as theaflavin and thearubigins can also impact the tea’s color (Morikawa et al., 2019). Therefore, FTLs constitute the material basis of the sensory qualities and health benefits of tea products. FTLs are also profoundly affected by a series of exogenous or endogenous factors, including artificial treatments during cultivation, geo-climate conditions, biotic and abiotic stresses, and genetic factors. From a practical perspective, shading treatment applied to tea plants in the field for an extended period could significantly alter the pigment content in chloroplasts, increase the accumulation of amino acids (including theanine), and decrease the contents of catechins and caffeine. Insects such as the leafhopper (*Empierce (Matsumurasca) onukii* Matsuda) activate the synthesis of volatile monoterpenes derived from linalool in infected tea leaves, such FTLs can be used to manufacture the famous oolong tea *Oriental Beauty* (Mei et al., 2017). In addition, the content of secondary metabolites can also vary significantly among cultivars, e.g., ‘Zijuan’ (*C. Sinensis* cv. Zijuan), a specialized cultivar with purple leaves, contains much more anthocyanins than ordinary cultivars (Zou et al., 2021). Thus, FTLs are an important medium connecting pre-harvest and post-harvest stages in the tea industry, which are mainly tea cultivation and processing. Based on this point of view, research in this field is highly valuable.

Recently, a series of reviews on several aspects of FTLs, including substance transformation during processing (Zhang et al., 2019), the synthesis and transport of theanine and catechins (Lin et al., 2022), (Liao et al., 2022), and the response of essential components under stress (Shao et al., 2021), have been conducted by researchers in this prospering field. However, it is still challenging to examine the development and changes of the entire field of research from a macro perspective, especially in terms of the evolution of research trends and future development. Bibliometric analysis is a systematic method that applies quantitative analysis and statistics to publications such as journal articles and their accompanying citation counts. It is helpful for researchers to analyze a research field related to a given scientific question from a global perspective, which can guide subsequent work. Over the past few decades, a set of bibliometric tools have been developed, among which the most used are Bibliometrix, CiteSpace, and VOSviewer. The integration of them has become a common approach in various fields, including medical field(Chen et al., 2022; Pei et al., 2022; Wan et al., 2022), biomaterials(Zheng et al., 2022), and food safety studies(Wei et al., 2022), due to their ability to provide more systematic and accurate information. Here, we attempted to quantitatively analyze the publication status, authorship collaboration, research hotspots, and trending topics of FTLs research using bibliometric methods, aiming in provide a comprehensive overview in this field.

## 2 Data source and analysis method

### 2.1 Data collection and screening

**Figure 1:**
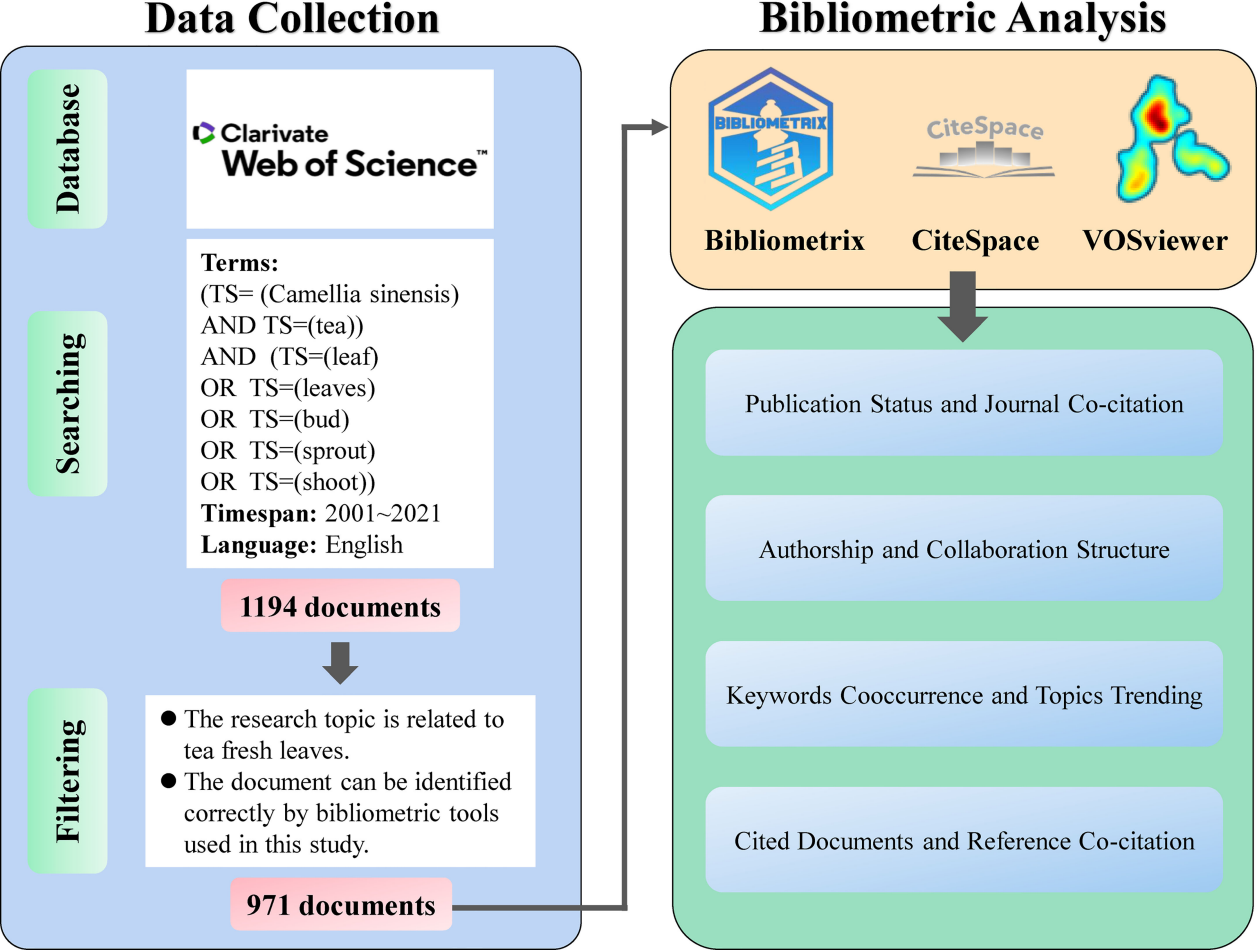
Data collection, filtering, and analysis of this study.

The Web of Science Core Collection (WoSCC) was chosen as the data source for the present study due to the completeness and high quality of its entries. Data retrieval was conducted on February 23, 2022, using [TS= (Camellia sinensis) AND TS=(tea)) AND (TS=(leaf) OR TS=(leaves) OR TS=(bud) OR TS=(sprout) OR TS=(shoot)] as the search term, with a timespan of publications from January 1, 2001, to December 31, 2021. The initial query yielded 1,957 publications, among which the document type was set to article, the language was restricted to English, and less relevant documents were removed *via* manual filtering. Next, 984 papers were retained for subsequent analysis, and 13 articles that Bibliometrix could not correctly identify were removed. A total of 971 qualified documents were subsequently used in this study. The conceptual design of the current work is highlighted in **Figure 1**.

### 2.2 Bibliometric analysis

Bibliometric analysis and visualization were performed using R package Bibliometrix (version 3.21), CiteSpace (version 5.8.R3-64bit), and VOSviewer (version 1.6.18). Microsoft Excel (version 2019) was used for data processing. Multiple units extracted from the dataset were used to conduct the bibliometric analysis, including journal, author, institution, country, reference, and keywords.

Two critical bibliometric metrics were referred (h-index and g-index) while estimating the scientific production and impact of top journals, authors, institutions, and countries. J.E. Hirsch proposed the h-index and defined it as the number of papers with citation numbers higher or equal to *h*; it is a helpful index used to characterize the scientific output of a researcher. The h-index considers the number of publications and citations received from individual documents and can be used to evaluate the impact of a scientific producer by avoiding the influence of individual highly and nominally cited papers (Hirsch, 2005). The g-index was introduced by Egghe in 2006, defined to representing top *g* papers that received at least *g^2^* citations, and it’s claiming to be a complete alternative to the h-index (Egghe, 2006). The g-index considers the citations of top documents, resulting in a more comprehensive view of one’s impact.

Networks used in science mapping usually include co-citation, co-occurrence, and collaboration networks. The Silhouette score (S score) and the centrality value are core indicators from CiteSpace and were applied to evaluate the consistencies of clusters and influence of nodes in each network. When the clusters of reference co-citation networks are generated, the S score is applied to validate cluster consistencies that range from −1 to +1. For example, a cluster with an S score of 1 usually indicates that the corresponding cluster is relatively isolated (Provart et al., 2016). Centrality measures the degree to which a node is in the middle of the path connecting other nodes in the network (Brandes, 2001). The higher its value, the more influential the node is generally, a value higher than 0.1 means that the node occupies an essential position in the network (Chen, 2012). It is typically represented as a purple circle in the network diagram, and the size of the circle is proportional to the centrality value. The detailed software usage and data processing methods are described in the **Supplementary Material**.

## 3 Results and discussion

### 3.1 Development stages of FTLs research

**Figure 2:**
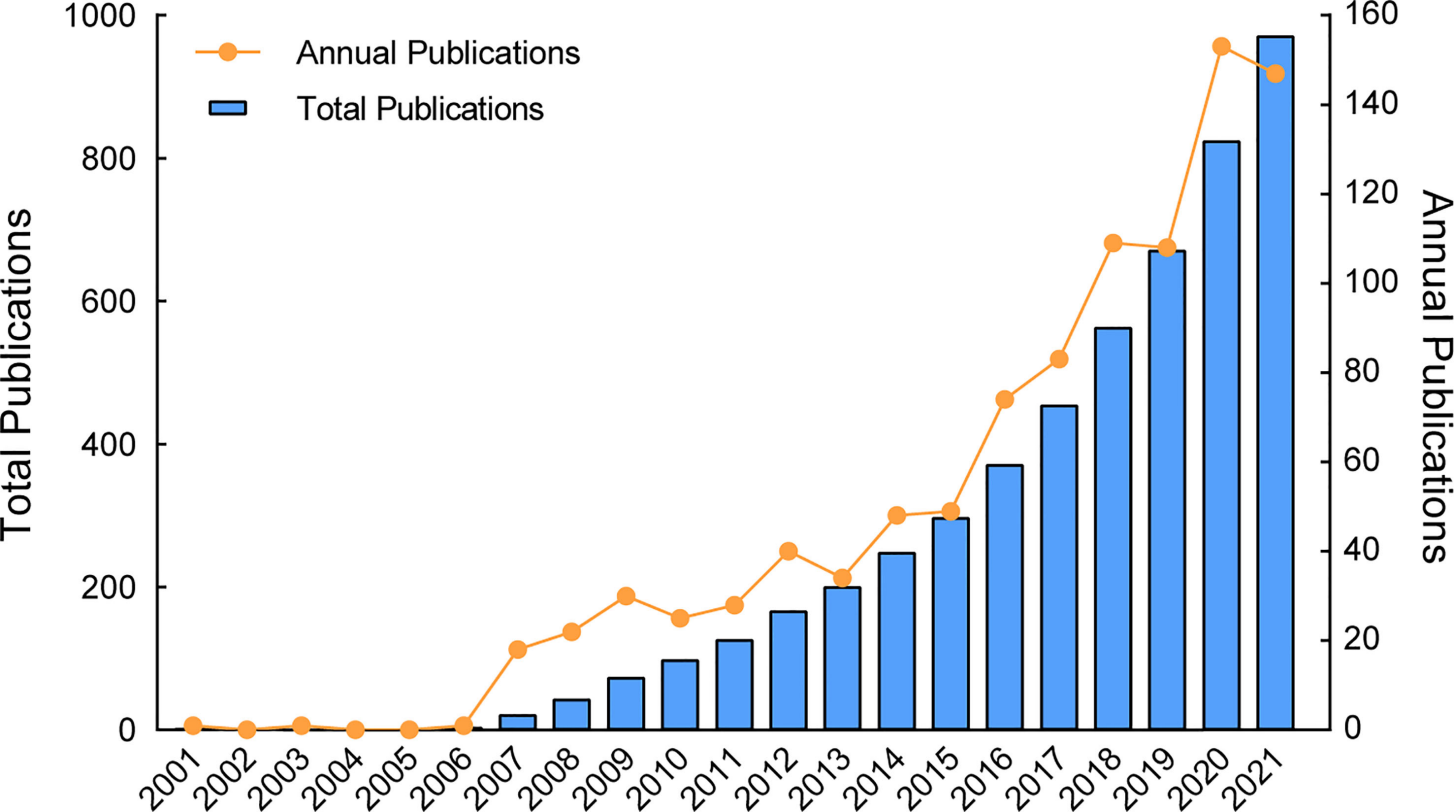
Total publications increment by time and annual publications of FTLs research from 2001 to 2021. The blue bars represent the accumulation of articles, while the orange line indicates the yearly scientific production.

After querying and data screening, 971 articles related to FTLs research were published between 2001 and 2021 (**Table 1**). The number of publications output has increased each year. However, it is worth noting that the number of papers published in 2021 is lower than in 2020, interrupting the increasing trend. Considering that there is usually a one- or two-year time lag between conducting experiments and paper publication, this deviation may be due to the impact of the COVID-19 epidemic, which could have hampered certain experimental processes in 2020. But certainly, FTLs research field has experienced a tremendous growth from scratch in the past two decades with an annual growth rate of 38.76% in total number of publications, as shown in **Figure 2**. The increase in total publications on FTLs research can be roughly divided into three stages based on the yearly scientific production, namely the initial stage (2001–2006), the slow development stage (2007–2015), and the rapid development stage (2016–2021), with 3, 294, and 674, publications in each stage, respectively.

**Table 1:** Main information of the data set used in this study after the manual screening.

Main information	
Timespan	2001-2021
Documents	971
Keywords	2529
References	27914
Countries	40
Institutions	762
Authors	2756
Journals	302
Language	English

There were only three papers were published in the initial stage and were mainly focused on how to isolate substances from FTLs and identify their structures (Ma et al., 2001; Inoue et al., 2003; Kobayashi et al., 2006). It was not until 2007 that many researchers started to focus on other topics related to FTLs. By the year of 2007, the Human Metabolite Database (HMDB) was created and was established formally (Wishart et al., 2007), given that the human genome, transcriptome, and proteome were already established. We speculate that the development stage in FTLs research is closely related to the establishment of metabolomics databases and the popularity of multi-omics technologies. The establishment of the metabolite database and the increasing advancement of metabolomics technologies may have facilitated the research about the secondary metabolite of FTLs. However, many scientific questions can hardly be solved with a single omics technique (i.e., the theanine synthetase gene was not identified until the first full genome assembly of tea plant was published) (Xia et al., 2017). Since then, many newly developed multi-omics techniques such as transcriptomics, proteomics, and genomics, which in turn accelerated the development in this field.

### 3.2 Core journals, authorship, and collaboration

#### 3.2.1 Core journals based on Bradford’s law

Bradford’s law describes the distribution of scientific publications in journals. It has been considered one of the fundamental laws of bibliometrics for decades and has been adopted by many researchers in bibliometric studies (Naranan, 1970; Venable et al., 2016). We analyzed the publication status and identified the core journals using Bradford’s law (**Figure S1**). The 971 articles were published in 304 journals, and the top ten most productive journals are listed in **Table 2**. These journals account for 3.31% (10) of the total journals and published up to 33.37% (29.87) articles. The top three highest publishing journals are *Journal of Agricultural & Food Chemistry*, *Frontiers in Plant Science*, and *Food Chemistry*, with 58, 39, and 36 published articles, respectively. The number of publications (NP), total global citation (TGC), h-index, categories, and citations per publication (CPP) of the core journal are also presented in **Table 2**. The TGC, h-index, and CPP of the *Journal of Agriculture and Food Chemistry* were 1075, 20, and 18.53, respectively. *Frontiers in Plant Science* ranked first in terms of TGC (1420) and h-index (23) and second in terms of CPP (36.41). Its NP was almost twice that of *BMC Plant Biology*, even though the latter holds the highest CPP value.

**Table 2:** Top ten produced journals with applied information (sorted by NP).

Journals	NP	TC	h-index	g-index	Categories	IF	CPP
Journal of Agricultural and Food Chemistry	58	1075	20	30	Q1, Agriculture	5.859	18.53
Frontiers in Plant Science	39	1420	23	37	Q1, Plant Sciences	6.627	36.41
Food Chemistry	36	833	16	28	Q1, Food Science & Technology	9.231	23.14
Scientific Reports	28	542	15	23	Q1, Multidisciplinary	4.996	19.36
Scientia Horticulturae	28	420	11	20	Q1, Horticulture	4.324	15.00
Plant Physiology and Biochemistry	26	505	16	22	Q1, Plant Sciences	5.437	19.42
BMC Plant Biology	21	770	14	21	Q1, Plant Sciences	5.260	36.67
International Journal of Molecular Sciences	21	246	11	14	Q1, Plant Sciences	6.208	11.71
Molecules	18	298	9	17	Q2, Multidisciplinary	4.297	16.56
Journal of the Science of Food and Agriculture	15	363	12	15	Q2, Multidisciplinary	4.125	24.20

NP, TC, IF, and CPP represent the number of publications, and total citations, impact factor, and citations per publication.

**Figure 3:**
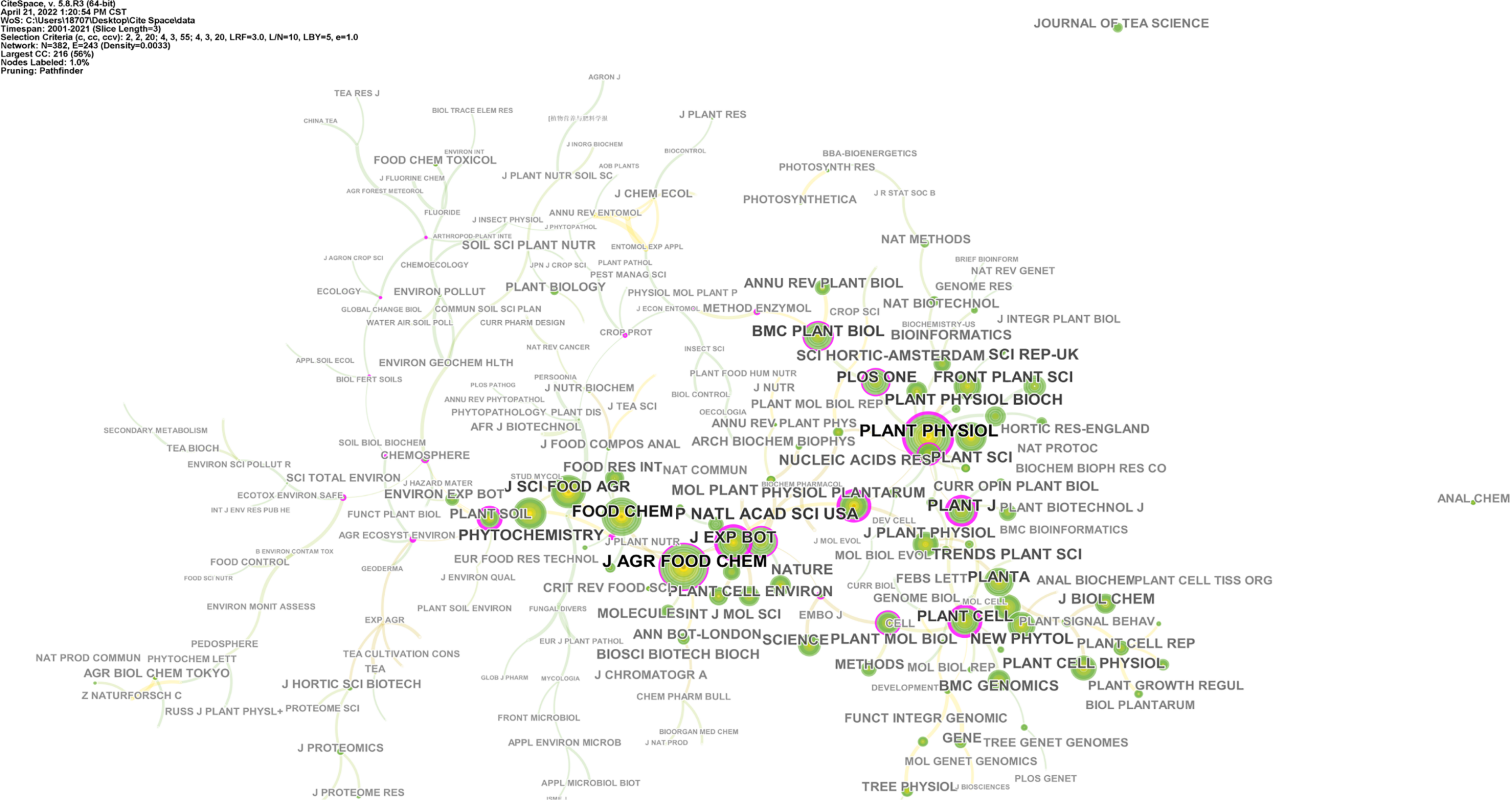
Journals co-citation network. The size of each node represents the citations of each journal. Colors closer to the middle of the node represent older publication years, whereas exterior colors are more recent publication years. The purple circle means the node’s centrality ≥ 0.1, and the size of the circle is proportional to the centrality value.

**Figure 3** shows the co-citation network of corresponding journals. Nodes with low production are set to a lower alpha value, resulting in higher transparency. Journals such as *Plant Physiology*, *PLOS ONE*, *Frontiers in Plant Science*, *BMC Plant Biology*, *Trends in Plant Science*, *The Plant Cell*, *New Phytologist*, and *Plant Molecular Biology* are related to plant science and are more closely distributed in the co-citation network. Other journals such as *Journal of Agriculture and Food Chemistry*, *Food Chemistry*, *Journal of Food Chemistry and Agriculture*, and *Phytochemistry* are related to food science and chemistry and share a closer relationship. The most co-cited journals include *Plant Physiology*, *The Plant Journal*, *The Plant Cell*, *BMC Plant Biology, Journal of Agriculture and Food Chemistry*, *Food Chemistry*, *Journal of the Science of Food and Agriculture*, and *Phytochemistry.* This reflects the multidisciplinary research context of the field.

#### 3.2.2 Scientific production and cooperation by country

**Figure 4:**
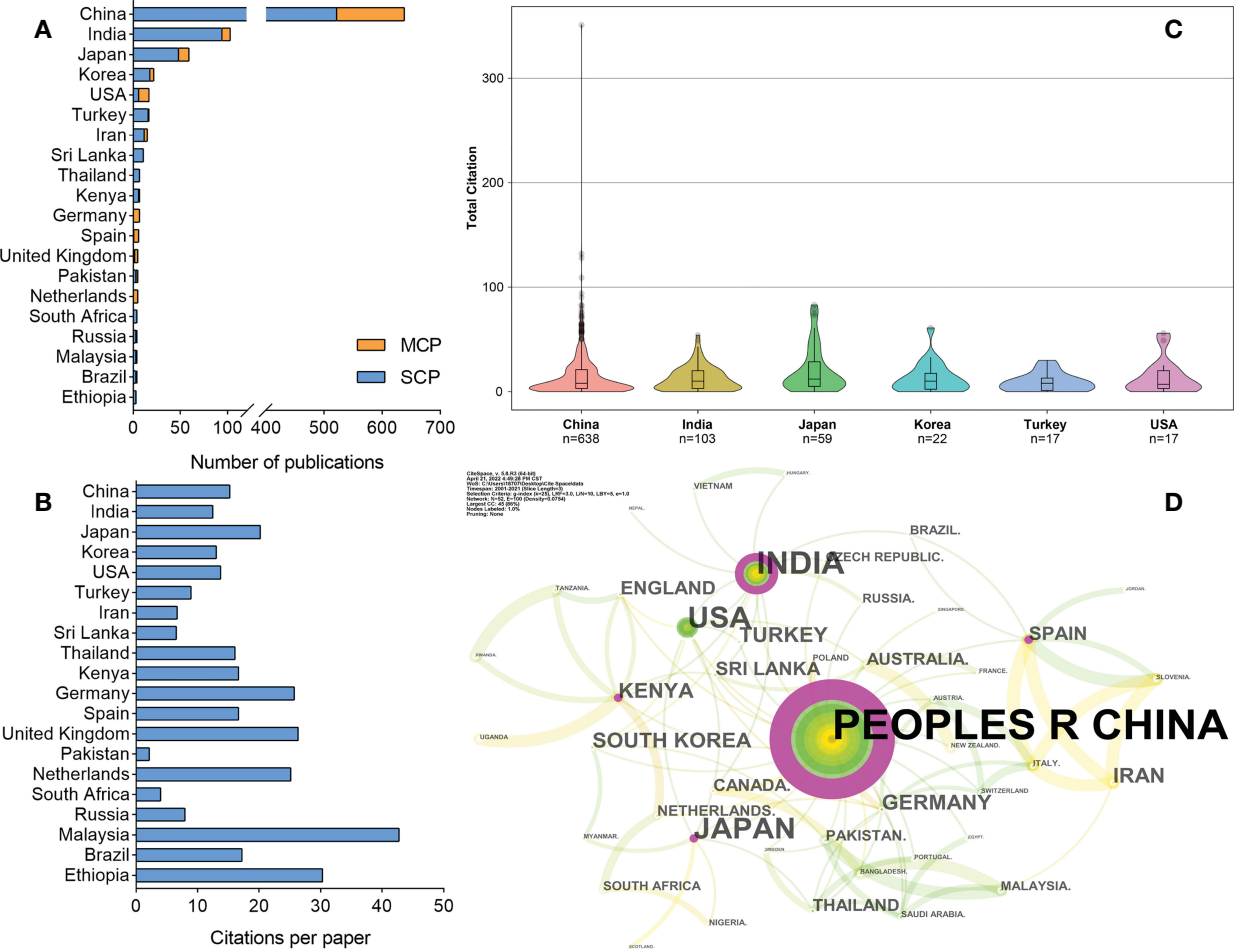
Countries’ scientific productions and collaboration relationship. **(A)** Scientific production and international collaboration by countries. **(B)** Citations per publications for each country. **(C)** The citation distribution of all papers from the top six countries. **(D)** Collaboration network of countries generated with CiteSpace. The size of each node represents the citations of each country. Colors closer to the middle of the node represent older publication years, whereas exterior colors are more recent publication years. The purple circle means the node’s centrality ≥ 0.1, and the size of the circle is proportional to the centrality value.

The tea plant is cultivated in the hilly and mountainous environments of tropical and temperate regions worldwide. Therefore, FTLs related research is of a global interest and the scientists involved in the contribution come from 40 countries worldwide. However, it is inevitably that the contribution among different counties vary, owing to a multitude of factors such as the distinct historical and cultural background of tea consumption, the variation in agricultural conditions, economic considerations, etc. In general, the countries with higher scientific production in this field are mainly located in Asia, North America, and Europe (**Figure 4A**), where Asia is the world’s major tea production and consumption region. In terms of this, China ranked first with 638 publications (i.e., 66% of the papers based on the corresponding author’s address) published in the past two decades and led in terms of total citations (9,745) (**Table S1**). China has thousands of years of tea-drinking history, and the country has developed a modern tea industry consisting of six different categories of tea: green tea, black tea, dark tea, white tea, oolong tea, and yellow tea. The Chinese government has placed great importance on the tea industry and the welfare of tea farmers, and some local governments have designated the tea industry as a key economic pillar industry. As a result, financial investment in tea science research has been increasing, leading to the strengthening of research efforts by universities and research institutions. These factors have contributed to the abundant research output in this field in China.The second most productive country is India, with 103 papers published. India is another country of the major tea producers and exporters, with a production and tea plant plantation area of 1.258 million tons and 637,000 hectares, respectively, which are second to China’s 2.986 million tons and 3.165 million hectares, respectively (Mei and Liang, 2022). Other major tea producers, such as Kenya, Sri Lanka, and Turkey, are also active in this field. However, many other countries such as Korea, the United States of America (USA), Germany, Spain, and Australia, also contributed to FTLs research and should not be ignored, despite they are not major producers. Korea and the USA feature in the list with 22 and 17 articles, respectively.

**Figure 4B** presents the CPP scores of the leading scientific nations in the field of FTLs. European countries, such as the United Kingdom, Germany, the Netherlands, and Spain, tend to exhibit relatively high CPP scores. In Asia, Japan stands out with the highest CPP among the prominent scientific producers, followed by China, South Korea, and India. Although Malaysia and Ethiopia also have high CPP scores, their publication counts are relatively low and are influenced more by a few highly cited publications. **Figure 4C** depicts the citation distribution of all papers from the six countries with the highest scientific output. Despite China’s contribution to some of the most highly cited papers in the field, the presence of a large amount of poorly cited literature drags down the average citation level. This may be linked to the rapid growth of Chinese research output in recent years (35% of output in 2021 and 2020), as newly published papers often require more time to accumulate citations from other researchers.

The cooperation relationships between different countries present in **Figure 4D**, a country collaboration map generated by Bibliometrix is presented in **Figure S2**. From the perspective of cooperation network, the most visible significant collaboration is the one between the USA and China, and a close cooperation sub-network exists among European countries. The ratio of multiple country production (MCP) and single country production (SCP) is related to international collaboration; we can use the percentage of MCP in the total number of publications to measure the functional level of international cooperation. The findings of this dataset (**Figure 4A**) showed that the countries with the highest cooperation rates were Germany (100%), the Netherlands (100%), Spain (83.33%), the USA (64.71%), and the United Kingdom (60.00%). It is worth noting that the rate of international cooperation is usually higher in European countries than in non-European countries, and higher in developed countries than in developing countries. However, China holds the highest value of centrality, indicating its essential role in the international collaboration network of FTLs research (**Figure 4C**, **Figure S2**).

#### 3.2.3 Scientific production and cooperation by institution

**Figure 5:**
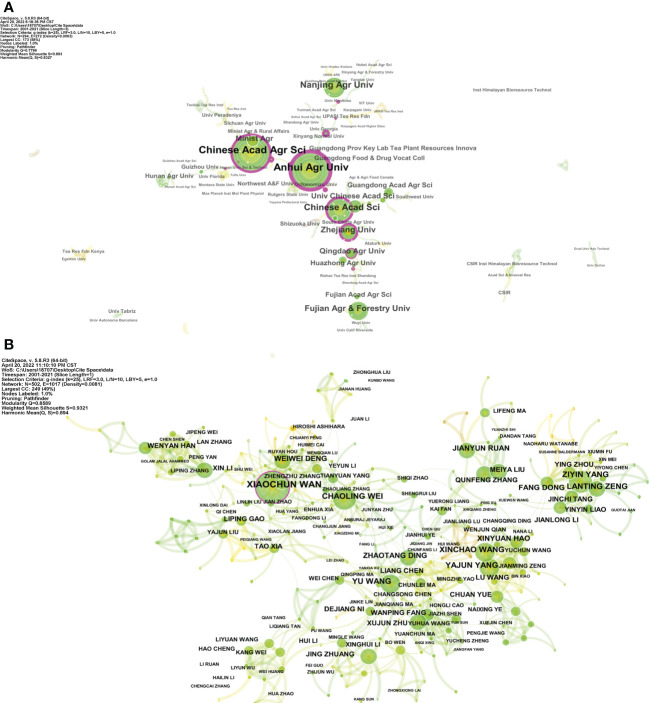
Collaboration network of **(A)** institutions and **(B)** authors, generated with CiteSpace. The size of each node represents the citations of each institution or author. Colors closer to the middle of the node represent earlier publication years, whereas exterior colors are more recent publication years. The purple circle means the node’s centrality ≥ 0.1, and the size of the circle is proportional to the centrality value.

Universities and public research institutes are the mainstays of FTLs research in terms of affiliated institutions, while private companies and research institutes do not appear as a key role. The top ten affiliated institutions are all located in China (**Table S2**), which may be related to China’s vast tea market and tea drinking culture. Among these, the Chinese Academy of Agricultural Science and Anhui Agricultural University hold a close publication, total citation, h-index, and centrality and have a substantial lead over Fujian Agriculture and Forestry University in third place. An institutional cooperation network describes the collaboration between institutions (**Figure 5A**), it shows that cooperation between research institutions is fragile. Affiliated institutions from different countries are scattered across the map and are not closely linked. Even though institutions in the same region cooperate considerably more frequently, there is still room for strengthening the cooperation.

#### 3.2.4 Scientific production and cooperation by author

Lotka’s law explains scientific productivity by determining the relationship between authors, and the quantities of their scientific outputs (Pao, 1985; Qiu et al., 2017). In the FTLs research field, 0.35% of total authors contributed 35.77% of the total quantity publications, basically, in line with the predicted trend of Lotka’s law (**Figure S3**). We further analyzed the top ten most published authors among them, their citations, h-index, and other related information are listed in **Table S3**. All the top ten authors are working in China, indicating a similar trend with the distribution of leading institutions. The top three productive authors with their publications, total citations (total global citations), and h-indexes are Xiaochun Wan (52, 1300, 18, respectively), Yu Wang (47, 435, 13, respectively), and Ziyin Yang (37, 858, 16, respectively), followed by Chaoling Wei (35, 1143, 18, respectively). According to the annual scientific production of top authors (**Figure S4**), some of them have been active in this field since 2007. Many others joined around 2009 and contributed to FTLs research development. By 2014, nine of the top ten authors were active in this field, and FTLs research began to enter the rapid development stage two years later.

Xiaochun Wan is a professor from Anhui Agricultural University, along with Chaoling Wei and Weiwei Deng. They are dedicated to studying tea plant biology, especially the biosynthesis of characteristic tea plant metabolites, such as catechins, theanine, caffeine, and anthocyanins, as well as their underlying regulatory mechanisms (Shi et al., 2011). Together, they produced the most highly cited paper in the current dataset by assembling a high-quality genome for the Chinese variation of tea plant (*C. sinensis* var. *sinensis*, CSS) (Wei et al., 2018) and established the Tea Plant Information Archive (TPIA, http://tpdb.shengxin.ren/), the first comprehensive web-accessible database designed for tea plants (Xia et al., 2019). Ziyin Yang and Lanting Zeng are affiliated with South China Botanical Garden, Chinese Academy of Sciences, and focus on researching the biosynthesis pathways and regulation networks of tea specialized secondary metabolites such as l-theanine (Cheng et al., 2017) and volatile compounds (Yang et al., 2012; Yang et al., 2013) in FTLs. A 2017 study found that the breakdown of chloroplast proteins may be necessary to accumulate free amino acids in FTLs under dark conditions (Chen et al., 2017), expanding our understanding of how cultivation practices such as shading treatment can improve tea quality.

We further explored the collaborative relationship between the authors (**Figure 5B**). We found that the trend of elitism is even more pronounced in terms of authorship than institutions. Many internally tightly connected clusters can be found within author collaboration networks, with the aggregation of these clusters coinciding with the authors’ affiliations. Authors were separated into multiple clusters in the collaboration network; these clusters showed close inner cooperation, whereas inter-cluster collaboration was relatively weak. For example, Xiaochun Wan, Chaoling Wei, and Weiwei Deng from the same institution are very close on the map. The same situation also applies to other authors such as Yu Wang with Zhaotang Ding, Ziyin Yang with Lanting Zeng, and Xinchao Wang with Yajun Yang. In addition, Xiaochun Wan possesses a higher centrality than other authors, hinting at his broad influence in the collaboration network of FTLs research.

### 3.3 Research trending topics

#### 3.3.1 Trending topics based on keyword co-occurrence and clustering

**Figure 6:**
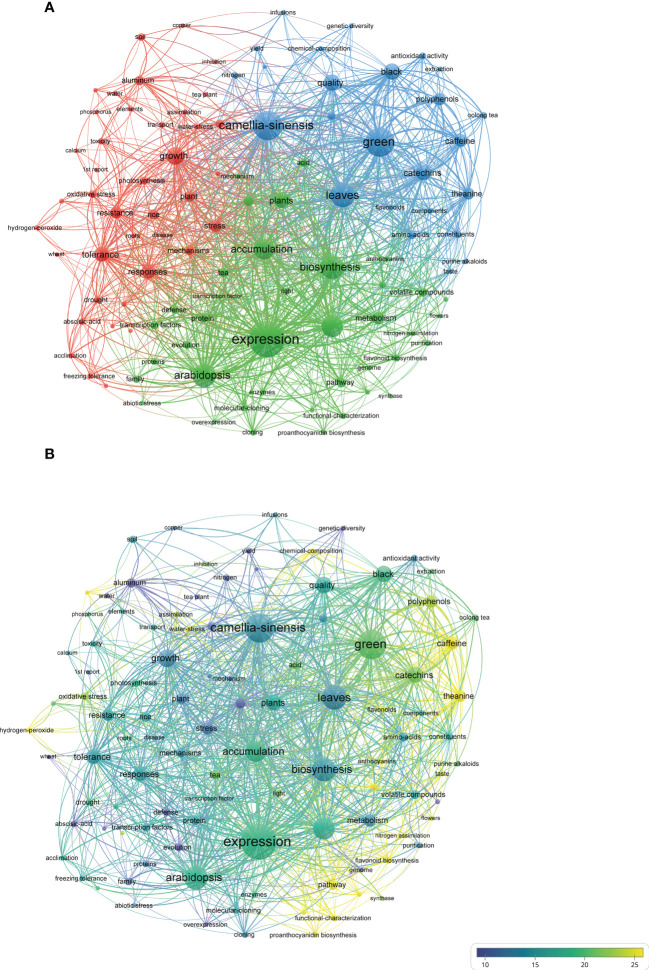
Co-occurrence network of keywords, generated with VOS viewer. The minimum number of occurrences is set as 10, resulting in 104 of all keywords meeting the threshold. The size of each node presents its event, and the co-occurrence network is colored by **(A)**: clusters and **(B)**: average citations.

Over the past twenty years of FTLs research, a considerable difference in the frequency of keywords was observed within the word cloud map (**Figure S5**). We also generated the keywords co-occurrence network using VOSviewer to further reveal the cluster relationship and co-occurrence status. One hundred and four keywords with a minimum occurrence of 10 were used to generate the plot (**Figure 6**). Among them, keywords used in data querying such as *camellia-sinensis* and *leaves* were excluded from this analysis.

Three clusters were identified using VOSviewer, namely green, blue, and red clusters (**Figure 6A**), that correspond to the different research topics, respectively. The average citations of keywords were also generated and shown in **Figure 6B**. Certain keywords with low frequency (e.g., *pathway*, *l-theanine*, *catechins*) received more citations. This finding might indicate that, although these topics have received widespread attention from researchers, the number of relevant studies still need to be improved. The more regularly cited keywords among the more frequently occurring group were *green*, *identification*, *genes (expression)*, and *arabidopsis*. These keywords are related to essential metabolites and their regulation mechanisms and are distributed in the green and blue clusters.

The green cluster is mainly related to the regulation mechanism of key genes, containing the highest frequency of the keyword *expression*. Other keywords with higher occurrence, such as *arabidopsis*, *accumulation*, *biosynthesis*, and *metabolism* are also classified into cluster green. The metabolism and characterization of essential compounds is the subject of the blue cluster, which includes the second most frequently occurred keyword, *green*. *Green* represents green tea, the most produced and consumed type among the six major types of tea products in China. Other types of tea, such as *black* and *oolong tea*, can also be found in this cluster. The keyword *quality* indicates the primary concern of this cluster. Other keywords of lower frequency, such as *catechins*, *caffeine*, *polyphenols*, and *theanine*, encompass the most important metabolites of the tea plant. The keywords in the red cluster were lower in frequency overall than the other two clusters but also conveyed rich information about the growth and stress responses of tea plants. The most common keywords in this cluster included *growth*, *tolerance*, *response*, and *stress*, which indicate the main interests of this cluster. More specific objects of study can be identified from less frequently occurring keywords, such as heavy and non-metallic elements such as *aluminum*, *cadmium*, *calcium*, *copper*, and *phosphorus*, and environmental factors such as *drought*, *freezing tolerance*, and *water*.

Keywords in the green cluster reflect the extensive interest of researchers in regulating gene expression in tea plants under various conditions. Researchers have identified essential genes in the pathway of several metabolites by applying genomics, transcriptomics, metabolomics, or combined multi-omics technologies, along with bioinformatics. For example, Xia et al. assembled the first high-quality genome of CSA and applied comparative transcriptomic analysis to obtain gene homologs related to the biosynthesis of *catechins*, *theanine*, and *caffeine* from 23 wild relatives (Xia et al., 2017). Further, they identified 13 genes encoding SAM-dependent N-methyltransferases (NMTs), which could catalyze the caffeine synthesis pathway from xanthosine, and found evidence to support the independent evolution of these genes. Keywords such as *arabidopsis*, *accumulation*, *biosynthesis*, *metabolism*, and *mechanism*, closely associated with *expression*, paint a comprehensive picture. Using *Arabidopsis thaliana* as an essential reference, researchers have uncovered a series of transcription factors in tea plants that play a crucial role in plant growth, stress response, and metabolite synthesis. For instance, *CsTCPs* can regulate stem tip development and catechin biosynthesis (Yu et al., 2021), *CsMYBs* were shown to be associated with l-theanine biosynthesis, and *CsMYB1* was further found to regulate trichome development and catechin synthesis in tea plants, applying bioinformatics and metabolomics technologies (Wen et al., 2020; Li et al., 2022a, 1). MYB transcription factor family genes can also be involved in the response of tea plants to drought and other stresses (Li et al., 2022b).

The quality of FTLs is closely related to the quality of processed tea products (Liao et al., 2022; Shao et al., 2021; Chaturvedula and Prakash, 2011). In the blue cluster, different types of tea and essential substances that determine tea quality were closely associated. *Green tea*, *black tea*, and *oolong tea* are the most popular tea products, while *catechins*, *caffeine*, and *theanine* are the most important substances. These FTLs substances and their corresponding processed teas have received much attention from researchers (Zeng et al., 2017; Zhou et al., 2017). The suitability of FTLs is fully discussed (Wang et al., 2019), and the quality changes of pre- and post-harvest fresh leaves were also explored (Fu et al., 2015). Moreover, agronomic practices were analyzed to determine their effect on the quality of FTLs and their finished tea (Arkorful et al., 2020; Chen et al., 2021; Rubel Mozumder et al., 2021).

The red cluster is related to tea plants’ growth and stress response and contains keywords representing heavy metals, non-metal elements, and other environmental factors. One of the most attractive characteristics of the tea plant is its hyper-enrichment in and tolerance to aluminum. In a comparative study with five other woody plants, the aluminum content of FTLs was 8,910 mg/kg, similar to *Melastoma affine* (9,930 mg/kg) but much higher than *Sterculia lanceolate* (26 mg/kg), *Ardisia crenata* (45 mg/kg), *Acacia Formosa* (58 mg/kg), and *Machilus thunbergia* (115 mg/kg) (Xie et al., 2001). Aluminum has recently been found to play an essential role in the growth and development of the tea plant root system (Sun et al., 2020); however, the health risk associated with a high aluminum content remains to be considered. Other metal elements, such as *cadmium* and *copper*, can also affect the growth and development of tea plants at different levels and pose potential food safety risks for tea products (Shu et al., 2003; Cao et al., 2010; Zhang et al., 2020a). Environmental factors such as *drought* (Lv et al., 2021; Ding et al., 2022), *freezing tolerance *(Yang et al., 2021), and *shade* (Wang et al., 2012a; Chen et al., 2017) have also been shown to affect the metabolic activities of FTLs and influence tea quality (Shao et al., 2021).

#### 3.3.2 The evolution of trending topics

**Figure 7:**
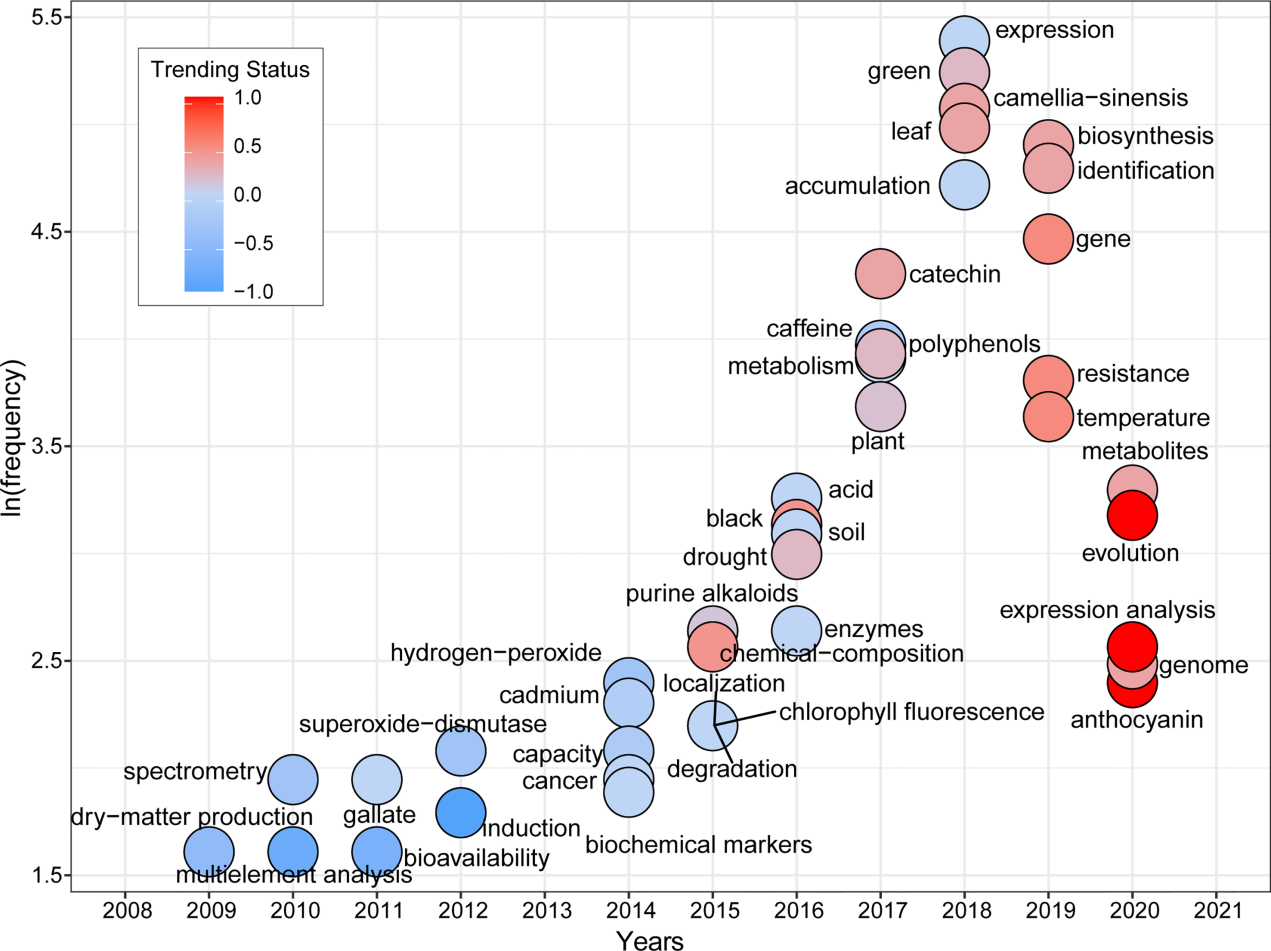
Trending topics in the field of FTLs research per year. A trending status value close to -1 means that the keyword was frequently mentioned in the early period of the trending duration and faded out over time. In contrast, a value close to +1 means that the keyword is increasingly popular. Due to the small quantity of publications before 2007, related keywords cannot be identified, detailed calculation method is described in the **Supplementary Material**.

**Figure 7** shows the temporal evolution of trending topics of FTLs-related studies over the past two decades, based on the keywords’ occurrence status. The definition of the trending status values, and related information are presented in **Table S4**. *Expression*, *green, black, biosynthesis*, *accumulation*, *catechin*, *theanine*, and other essential metabolites of FTLs had higher frequency and are higher in the value of trending status, demonstrating their continued focus in the field. From 2009 to 2015, trending topics included *dry-matter production*, *spectrometry*, *superoxide-dismutase*, *hydrogen-peroxide*, and *purine alkaloids*, leaning more towards basic biochemical and enzymatic studies. Around 2016, the interest in the field rapidly expanded to include tea quality, metabolite biosynthesis, and tea plant stress response. Keywords such as *soil*, *plant*, *chemical composition*, and *chlorophyll fluorescence* appeared in 2015 and 2016, representing the influence of environmental factors on tea plants and corresponding to the red cluster in **Figure 6A**. The median year for the keywords *catechin*, *caffeine*, and *polyphenols* was 2017. These keywords correspond to the blue cluster, reflecting the focus on essential compounds and the quality of tea products during this period. In the last four years of the two past decades, trending topics such as *expression*, *biosynthesis*, *identification*, and *gene* represent the intense focus on the regulatory mechanisms underlying key gene function. Moreover, it is worth noting that *evolution* and *genome* appear in 2020, with a relatively high frequency and trending status value, indicating that these keywords have gained much attention in a short period around 2020. Another recently trending topic is *anthocyanin*.

### 3.4 Hotspots based on publication and reference co-citation

#### 3.4.1 Most influential publications

Bibliometrix was used to identify the most locally and globally cited publications, according to the cited times of each publication, the related information of most influential publications are presented in **Table 3**.

**Table 3:** Top five most cited articles locally and globally.

Title	(Authors, publication year)	Source	LC (rank)	GC (rank)	Keywords
Draft genome sequence of *Camellia sinensis* var. *Sinensis* provides insights into the evolution of the tea genome and tea quality.	(Wei et al., 2018)	Proceedings of the National Academy of Sciences	150 (1)	351 (1)	Comparative genomics; genome evolution; catechins biosynthesis; theanine biosynthesis; tea quality
Determination of quality constituents in the young leaves of albino tea cultivars	(Feng et al., 2014)	Food Chemistry	43 (2)	81 (12)	*Camellia sinensis*; albino tea; metabolic profiling; multivariate analysis; tea quality
Tissue-Specific, Development-Dependent Phenolic Compounds Accumulation Profile and Gene Expression Pattern in Tea Plant [*Camellia sinensis*]	(Jiang et al., 2013)	PLOS ONE	42 (3)	132 (3)	MYB transcription factor; green tea; pro-anthocyanidin biosynthesis; anthocyanidin-reductase; flavonoid biosynthesis; Medicago-truncatula; ectopic expression; seed coat; *Arabidopsis*; catechins
Characterization of volatile and non-volatile metabolites in etiolated leaves of tea (*Camellia sinensis*) plants in the dark	(Yang et al., 2012)	Food Chemistry	40 (4)	83 (10)	Capillary electrophoresis–time of flight mass spectrometry; Dark; Etiolation; Metabolite; Tea; Volatile
Influence of shade on flavonoid biosynthesis in tea (*Camellia sinensis* (L.) O. Kuntze)	(Wang et al., 2012a)	Scientia Horticulturae	38 (5)	109 (5)	Catechins Flavonoid metabolism; Gene expression; O-glycosylated flavanols; Proanthocyanins; Shade Tea
Antioxidant activity of *Camellia sinensis* leaves and tea from a lowland plantation in Malaysia.	(Chan et al., 2007)	Food Chemistry	3 (264)	158 (2)	*Camellia sinensis*; Fresh leaves; Tea; Lowland; Highland; Total phenolic content; Antioxidant activity
*De novo* assembly and transcriptome characterization: novel insights into catechins biosynthesis in *Camellia sinensis*	(Wu et al., 2014)	BMC Plant Biology	0 (-)	128 (4)	*Camellia sinensis*; Transcriptome; High-throughput sequencing; Catechins; RP-HPLC; Genetic diversity

TLC and TGC represent totally local citations and totally global citations, respectively.

The highest locally cited paper (150 times) yet was published by Professor Chao-Ling Wei in 2018; this number is much higher than for other papers (Wei et al., 2018). It is also the most globally cited publication (351 times) in the dataset. In this study, Wei et al. assembled the first high-quality genome of the Chinese variety of tea plant. This genome is 3.14 Gb in size and has scaffold contig N50 of 1.39 Mb. A comparison of the CSS genome with the first published Assam-type tea plant (*C. sinensis* var. *assamica*) (Xia et al., 2017) showed that the differentiation time was approximately 0.38–1.54 million years ago between the two tea plants varieties. A whole-genome duplication (WGD) event that occurred in the ancestral species of *C. sinensis* was further identified; it is believed that this event contributed significantly to the origins of critical genes in the biosynthesis pathways of catechins. The authors also identified the glutamine synthetase (GS) gene *CsGSI* as the candidate gene for theanine synthesis (TS) and thus renamed it *CsTSI*.

Albino or etiolated tea plants garnered much interest among FTLs researchers over the past two decades, having been the focus of two of the five most locally cited papers. Feng L systematically determined the quality components in young leaves and found that three albino tea plants had significantly higher amino acid content and substantially lower chlorophyll, catechin, and caffeine content than normal plants (Feng et al., 2014). Tea plants with such phenotypes were often considered suitable for producing high-quality green tea (Zhang et al., 2020d). Professor Yang Ziyin’s research also investigated tea plant resources by observing their physiological responses under dark conditions and the changes in volatile and non-volatile metabolites ((Yang et al., 2012).

The remaining two of the most locally cited publications reported on the biosynthesis and accumulation mechanisms of flavonoids in the tea plant. We noticed that Tao Xia was the common responding author of the two papers. The team first investigated the differences in flavonoid contents of tea plants under shading treatment and regular light. They found that the contents of lignans, catechins, and proanthocyanins decreased significantly, whereas the contents of phenolic acids increased (Wang et al., 2012a). In a study published in 2013, they further explored the accumulation of metabolites in the shikimic, phenylpropanoid, and flavonoid pathways in different tissues of the tea plant and investigated the expression profiles of related genes (Jiang et al., 2013). In addition, shade treatment — a commonly applied cultivation regimen — was a highlight among the top-cited publications, with two related papers featured in the list (Wang et al., 2012a; Yang et al., 2012).

Our analysis of the most cited publications showed that researchers are likely to pay more attention to studies with the following highlights.

*Studies that introduce new technologies.* Multi-omics techniques have been widely applied in the study of FTLs. Researchers have used metabolomics techniques to identify the major substances in FTLs that contribute to flavor (Zhou et al., 2017) and color (Shen et al., 2018). They also explored the changes in the accumulation of these substances under different conditions in depth (Liao et al., 2022). Metabolomics techniques have also helped researchers understand key metabolites’ dynamics during tea processing (Yu et al., 2020; Ye et al., 2022). The application of transcriptomics has dramatically deepened the understanding of the synthesis and regulation mechanism of key metabolites (Yu and Yang, 2020), the mechanism of the tea plant’s adversity response (Zeng et al., 2019), the mechanism of tea plant mutant formation (Zhang et al., 2020b), etc. The most locally and globally cited paper was contributed by Wei et al., 2018, who assembled the first high-quality reference genome of CSS. This work is foundational to future studies in tea plant genetics, domestication, and transcriptomic studies. The availability of reference genomes allows for transcriptomes with reference genomes and large-scale resequencing studies to be carried out (Wang et al., 2020; Lu et al., 2021), and for researchers to conduct genome-wide gene family screening and functional studies more efficiently (Wang et al., 2018). In recent years, in addition to the rapid emergence of genomic technologies based on genome-wide association study (GWAS) and metabolome genome-wide association study (mGWAS) in FTLs research have become new research paradigms, the combined use of multi-omics technologies to comprehensively researched the FTLs field is also a new wave in the field of research. A recent study has shown that structural variants identified based on pangenome are valuable in designing maker assays (Zhou et al., 2022); however, pangenome research on tea plants remains non-existent. The development of plant pan-genome may bring essential opportunities for FTLs research.

*Studies that applied leaf color variants*. Markedly altered levels of essential metabolites usually accompany leaf color variants such as albino or purple leaves. The occasional occurrence of albino individuals among tea plants was discovered early on, and numerous attempts have been made to explore the regulatory mechanisms of albinism. The first reported albino tea cultivars were ‘Xiaoxueya’ and ‘White leaf No.1’ (also known as ‘Baiye No.1’ in recent studies), whose leaves turn yellow under low temperatures (Du et al., 2006). The number of reported albino tea plant variations has dramatically expanded, with a recent review from China listing over 50 (Liu et al., 2020). Researchers have found that albino tea plants could be classified as light-sensitive, temperature-sensitive, and ecologically insensitive (Zhang et al., 2020c). Their chloroplast development (Gao et al., 2021), leaf structure (Du et al., 2008), metabolic profiles (Li et al., 2015a), and gene expression profiles (Liu et al., 2022) were also discussed in various publications. Purple leaf variations, such as ‘Mooma1’, are highly accumulated with anthocyanins, resulting in unique appearances and flavor. They also constitute valuable samples for studying flavonoid-related pathways (He et al., 2018). These materials are crucial for studying the metabolism and regulatory mechanisms of related substances and have considerable economic value because of their distinctive qualities. However, the mechanism underlying the formation of different albino tea plants remains unclear.

*Critical studies on essential metabolites*. Keyword co-occurrence, top-cited papers, and trend topics proved that essential metabolites are the core topics of FTLs research. The metabolic pathways and their contribution to the quality of theanine, catechin, and caffeine have been determined. However, their role in the life activities of tea plants has not been fully demonstrated. An established mechanism is that theanine is synthesized mainly in the roots of tea plants and transported to the leaves. Therefore, theanine will likely act as a nitrogen carrier for long-distance transport. Polyphenols are active components in the oxidative stress response of tea plants and are considered indicators for selecting drought-tolerant cultivars (Cheruiyot et al., 2007). Nevertheless, the role of catechins — a unique substance and major polyphenolic component in tea plants — beyond antioxidants remains unclear. In recent years, anthocyanins and proanthocyanins, which are closely related to the metabolic pathway of catechins, have also received increasing attention.

*Studies in which applied cultivation treatments affected the essential metabolites*. Cultivation treatments can affect the chemical composition of fresh tea leaves. For example, the most widely used shade treatment can increase the amino acid content, reduce the flavonoid content, and change the color of the fresh leaves of tea plants (Ku et al., 2010; Lee et al., 2013). Moreover, recent studies have shown that appropriate drought treatment can enhance theaflavin accumulation in tea plant leaves (Lv et al., 2021). These findings hint at a promising future — one in which the collection of different natural products can be increased using simple, low-cost cultivation measures to meet the needs of different production types.

#### 3.4.2 Hotspots’ evolution based on trends in cited references

**Figure 8:**
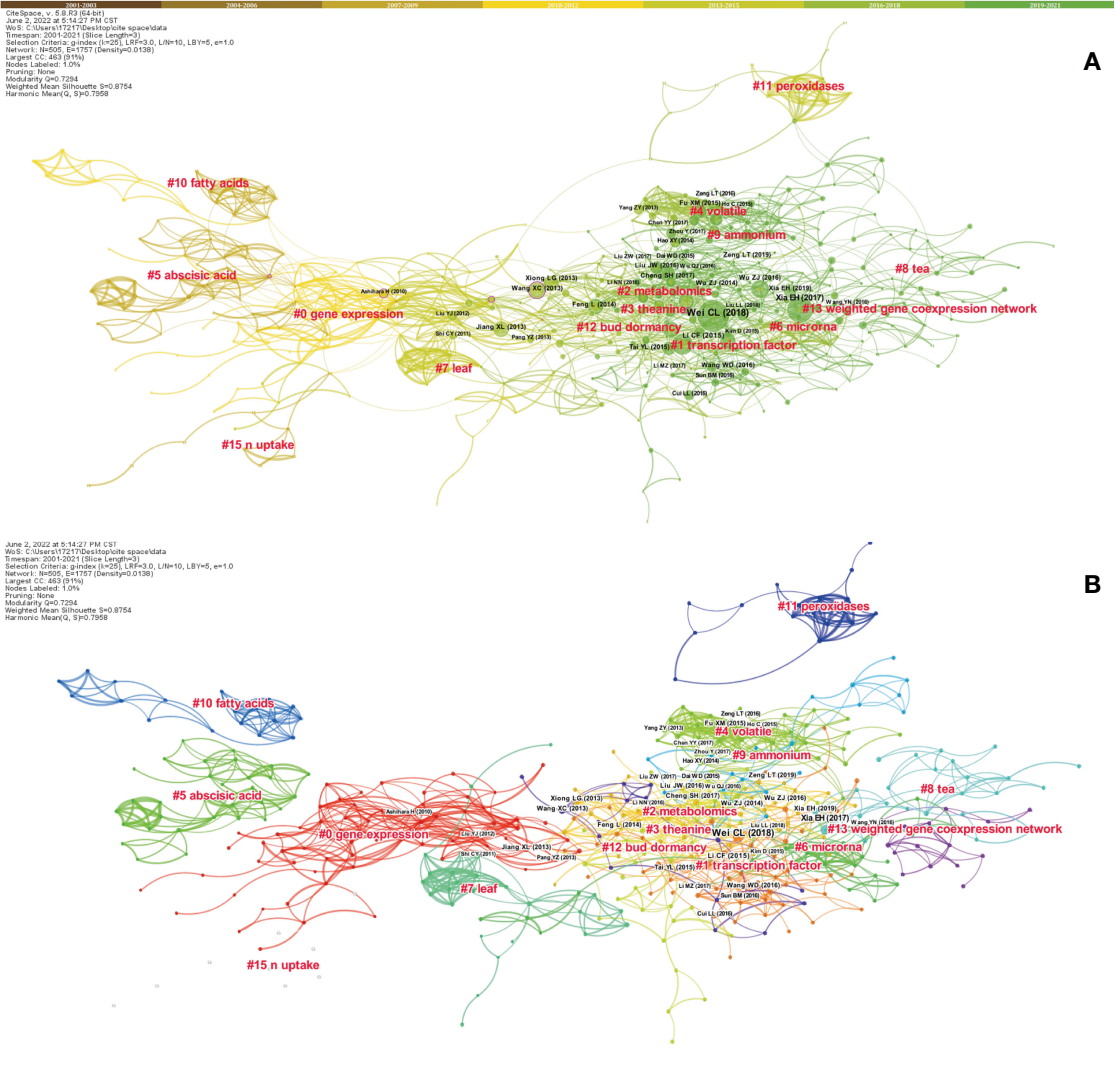
Co-citation references network (1980–2021) and correspondent clustering analysis obtained with CiteSpace. **(A)** Co-citation reference network with cluster visualization. The size of each node represents the citations of each reference. Colors closer to the middle of the node represent older publication years, whereas exterior colors are more recent publication years. The purple circle means the node’s centrality ≥ 0.1, and the size of the circle is proportional to the centrality value. **(B)** Visualization map of the corresponding clusters.

Cited references can be considered the intellectual base of a particular research front (Chen, 2006). We applied CiteSpace to construct the co-citation network of the cited references, aiming to explore the theoretical base of FTLs research (**Figure 8**). These references were clustered into 15 clusters. The size, S score, and average year of publication of collections are satirized in **Table S5**, and the co-citation networks for each time slice (three years) are presented in **Figure S6**. Four trends were found based on a comprehensive analysis of the above information.

The first trend appeared around 2005. We describe clusters in terms of the number of publications, S score, and average publication year, shown within brackets. The clusters contained in this trend were #5 *abscisic acid* (32, 0.981, 2005), #15 *n uptake* (9, 0.983, 2005), #10 *fatty acid* (18, 0.989, 2006), #0 *gene expression* (69, 0.787, 2009), #7 *leaf* (26, 0.953, 2011) and #12 *bud dormancy* (16, 0.915, 2012). Cluster #11 *peroxidases* (17, 0.991, 2012) was a relatively isolated cluster, representing a series of parallel studies that failed to merge with the mainstream research and was also part of trend one, based on the temporal perspective. The second and third trends both started from clusters #0, #7 and #12, and shared clusters #2 *metabolomics* (47, 0.868, 2015) and #3 *theanine* (42, 0.842, 2015). Trend two further developed into cluster #4 *volatile* (38, 0.924, 2016), and trend three also contained cluster #9 *ammonium* (21, 0.929, 2017). Trend four started from clusters #2 and #3, developed into clusters #1 *transcription factor* (0.747, 2016), #8 *tea* (25, 0.896, 2017), and #13 *weighted gene co-expression network* (15, 0.916, 2019), joined by cluster #6 *microRNA* (29, 0.888, 2014).

Key references with high centrality were identified using CiteSpace (**Table S6**). For example, Mamati’s article (Mamati et al., 2006) was essential in transforming from cluster #5 *abscisic acid* to cluster #0 *gene expression*. Cluster #0 also included many other key references, including (Eungwanichayapant and Popluechai, 2009; Ashihara et al., 2010; Wang et al., 2012b; Deng et al., 2013). Among these, (Wang et al., 2012b) had a close co-citation relationship with (Wang et al., 2013) from #12 *bud dormancy* and “Fan et al. (2015)” ^47^ from #9 *ammonium*. It should be noted that “Wang et al. (2013)” held an important position in the co-citation network as the first key reference of the rapid development stage, as well as the only one that was co-cited with (Hao et al., 2014).

The trending topics and reference co-citation network reflected the development and evolution of the research hotspots and interests in the field of FTLs research. During the initial stage, the number of publications was too low to identify meaningful keywords. By contrast, the topics in this field were significantly expanded during the slow development stage. Researchers started with biomass production, as well as physiological and enzyme-related topics, and quickly progressed to studies on tea plant stress response and essential metabolites. With the increasing number of publications, the regulatory mechanisms underlying the biosynthesis of important compounds were important concerns during the rapid development stage. Keywords related to tea quality such as *amino acids*, *caffeine*, *polyphenols*, and *catechins* have received considerable attention over the last two decades. Keywords such as *genome* and *evolution* have rapidly become popular topics in the previous two or three years, in connection with the recent release of a series of high-quality genome assemblies of tea plants (Wang et al., 2021; Zhang et al., 2021).

However, this does not represent a competitive relationship between the new omics technologies and other topics. On the contrary, as Xia pointed out (Xia et al., 2020), the high-quality genome assembly of different plant types greatly enhanced our understanding of the molecular mechanisms underlying tea quality to produce theanine (Wei et al., 2018), caffeine (Xia et al., 2017), and aromatic compounds (Wang et al., 2021). Trends in the intellectual base reveal similar patterns. Trend one indicates the interest in fundamental biochemical and enzymatic studies at the beginning of the slow development stage, while gene expression in tea plants was widely noted (Mamati et al., 2006; Eungwanichayapant and Popluechai, 2009). After years of exploration, with the metabolic pathways of the essential substances largely elucidated, the researchers’ interest quickly focused on crucial metabolites such as theanine (Deng et al., 2013). Three differentiating trends emerged in a short period. Trends two and three focused on tea quality and tea plant physiology, whereas trend four represented a considerable increase in the application of molecular biology methods in the FTLs research field, as demonstrated by the cluster titles *transcription factor*, *microRNA*, and *weighted gene co-expression network*.

## 4 Conclusions and perspectives

This study analyzed the stages of development, authorship collaboration, research topics, and hotspots and their temporal evolution trends in the past 20 years of the field of fresh tea leaves, applying bibliometric tools. This result showed that the field has experienced a rapid growth in the past two decades, with more than 100 articles published annually. The development history was divided into three stages based on the publication status and research topics, namely initial stage, slow development stage and rapid development stage. Most influential journals were identified by Bradfords’ law, among which *Journal of Agricultural & Food Chemistry*, published most of the articles and *Frontiers in Plant Science* holds the highest total citations as well as h-index. The most influential countries, institutions, and authors were also identified as China, Chinese Academy of Agricultural Science and Xiaochun Wan, respectively. Collaboration between authors from different institutions was found significantly less than that within the same institution. Since it has been well documented that scientific collaboration facilitates increased scientific output and impact across regions or countries, establishing broad and efficient cooperation will be a challenge in driving this area forward in the future.

Researchers have extensively explored three themes within the field, namely, the regulatory mechanism of essential genes, the metabolism and features of crucial compounds, and the growth and stress responses of tea plants, and have made some progress. These research topics emerged in 2009 and have maintained a blooming development trend. The introduction of new technologies and multi-omics approaches, the investigation of leaf color variants, the examination of essential metabolites, and the development of cultivation practices to enhance tea quality have been given significant attention in the field. The cited references in this field have formed 15 clusters and four evolutionary trends, which also confirmed the evolution of research topics.

Based on the results of our analysis, the exploration on following areas is expected to be emerging hotspots in the coming years and will contribute to the development of this field.

1) To utilize a variety of tea germplasm, including wild populations, wild relatives, cultivars, and special variants, to advance the progress of tea breeding. High-quality varieties are the foundation for satisfying the demand for premium FTLs and diversifying tea product markets. However, numerous challenges persist in the field of tea breeding. Despite the development of molecular markers for critical traits in tea plants, their practical use in breeding programs is limited by the large genome size, self-incompatibility, high heterozygosity, and absence of stable gene transformation systems in tea plants. Currently, most commercial varieties are produced through selective breeding from wild populations and elite variants. These selective breeding methods, however, are proving challenging as a basis for continuous genetic improvement. Hence, new materials and methods are required to enhance the existing breeding system. The identification of low-heterozygous germplasm, overcoming barriers to self-incompatibility, and establishing interspecific hybridization systems are crucial steps towards modernizing tea breeding programs. And the extensive range of wild tea populations in East and Southeast Asia, wild relatives, and various characterizing variants (e.g., albino and purple leaf mutants) present during cultivation offer diverse materials for these efforts. Recent advancements in molecular biology techniques, such as the integration of transcriptomic, genomic, and epigenetic techniques, and the eventual assembly of tea plant pan-genome, will aid researchers in identifying QTLs (Quantitative Trait Loci) for crucial traits, such as resistance and quality, on a larger scale. Although many pre-works are required, such as the refining of reference genomes, improvements in breeding systems can significantly enhance the quality of FTLs, helping operators mitigate the risks associated with global climate change and market volatility.
2) Exploring the stress response patterns of tea plant specialized metabolites have the potential to open new avenues for improving cultivation techniques to producing high-quality FTLs. Typically, unexpected stress leads to negative impacts on tea plant growth and yields. However, recent studies have demonstrated that fluctuations in essential metabolite levels under various stress conditions can bring improvements in the quality of FTLs. For instance, tea plants exposed to drought or high temperatures often show an increase in catechin content (Wang et al., 2016), while shading conditions lead to increased levels of amino acids (Shao et al., 2022). Low temperature conditions have also been found to increase anthocyanin content (Han et al., 2017). Furthermore, it is already well known that FTLs harvested after being exposed to multiple stressors have a more intense aroma. In the context of the continuous growth of global tea production, research into fine-controlling artificial stress treatments in production practices will be crucial in the pursuit of high-quality FTLs. Techniques like shading through covering and intercropping, artificial drought, and the targeted intervention of phytophagous insects can be used to deliberately increase the content of desirable metabolites, leading to the tea products with enhanced taste, aroma, or active compounds.
3) To establish a unidirectional regulatory system that correlates quality traits in FTLs with tea products, researchers need consider multiple stages of the tea industry, including breeding, cultivation, processing, and quality evaluation. While various theoretical studies have accumulated on the variation of quality components of FTLs under different growth environments and artificial cultivation practices. Also, changes in metabolites, enzyme activities during tea processing, the effect of processing techniques and agents on the flavor of tea products, and the flavor characteristics of different tea types have been studied. The major challenge at present is the ineffective application of research findings at the pre- and post-harvest stages to production practice. To fill this gap, a unidirectional regulatory system is needed to regulate cultivation practices and produce high-quality FTLs for processing into premium tea products. Future researchers should view tea production as a system project and enhance the applicability of research results by utilizing multi-stage experimental designs.

## Author contributions

YQC and YFL worked together to conceptualize the paper, collect, and analyze the data and write the manuscript. CWS and LZX guide the work and provide the support. All authors contributed to the article and approved the submitted version.

## References

[B1] Arkorful E. Yu Y. Chen C. Lu L. Hu S. Yu H.. (2020). Untargeted metabolomic analysis using UPLC-MS/MS identifies metabolites involved in shoot growth and development in pruned tea plants (Camellia sinensis (L.) o. kuntz). Scientia Hortic. 264, 109164. doi: 10.1016/j.scienta.2019.109164

[B2] Ashihara H. Deng W.-W. Mullen W. Crozier A. (2010). Distribution and biosynthesis of flavan-3-ols in camellia sinensis seedlings and expression of genes encoding biosynthetic enzymes. Phytochemistry 71, 559–566. doi: 10.1016/j.phytochem.2010.01.010 20189205

[B3] Brandes U. (2001). A faster algorithm for betweenness centrality. J. Math. Sociology 25, 163–177. doi: 10.1080/0022250X.2001.9990249

[B4] Cao H. Qiao L. Zhang H. Chen J. (2010). Exposure and risk assessment for aluminium and heavy metals in puerh tea. Sci. Total Environ. 408, 2777–2784. doi: 10.1016/j.scitotenv.2010.03.019 20413147

[B5] Chan E. W. C. Lim Y. Y. Chew Y. L. (2007). Antioxidant activity of camellia sinensis leaves and tea from a lowland plantation in Malaysia. Food Chem. 102, 1214–1222. doi: 10.1016/j.foodchem.2006.07.009

[B6] Chaturvedula V. S. P. Prakash I. (2011). The aroma, taste, color and bioactive constituents of tea. JMPR 5, 2110–2124. doi: 10.5897/JMPR.9001187.

[B7] Chen C. (2006). CiteSpace II: Detecting and visualizing emerging trends and transient patterns in scientific literature. J. Am. Soc. Inf. Sci. Technol. 57, 359–377. doi: 10.1002/asi.20317

[B8] Chen C. (2012). Predictive effects of structural variation on citation counts. J. Am. Soc Inf. Sci. Technol. 63, 431–449. doi: 10.1002/asi.21694

[B9] Chen Y. Fu X. Mei X. Zhou Y. Cheng S. Zeng L.. (2017). Proteolysis of chloroplast proteins is responsible for accumulation of free amino acids in dark-treated tea (Camellia sinensis) leaves. J. Proteomics 157, 10–17. doi: 10.1016/j.jprot.2017.01.017 28163235

[B10] Chen J. Liu J. Liu X. Zeng C. Chen Z. Li S.. (2022). Animal model contributes to the development of intracranial aneurysm: A bibliometric analysis. Front. Vet. Sci. 9. doi: 10.3389/fvets.2022.1027453 PMC9716216 36467643

[B11] Chen Y. Wang F. Wu Z. Jiang F. Yu W. Yang J.. (2021). Effects of long-term nitrogen fertilization on the formation of metabolites related to tea quality in subtropical China. Metabolites 11, 146. doi: 10.3390/metabo11030146 33801425 PMC8000315

[B12] Cheng S. Fu X. Wang X. Liao Y. Dong F. Yang Z. (2017). Studies on the biochemical formation pathway of the amino acid l -theanine in tea ( camellia sinensis ) and other plants. J. Agric. Food Chem. 65, 7210–7216. doi: 10.1021/acs.jafc.7b02437 28796499

[B13] Cheruiyot E. K. Mumera L. M. Ngetich W. K. Hassanali A. Wachira F. (2007). Polyphenols as potential indicators for drought tolerance in tea (Camellia sinensis l.). Biosci. Biotechnol. Biochem. 71, 2190–2197. doi: 10.1271/bbb.70156 17827703

[B14] Deng W.-W. Fei Y. Wang S. Wan X.-C. Zhang Z.-Z. Hu X.-Y. (2013). Effect of shade treatment on theanine biosynthesis in camellia sinensis seedlings. Plant Growth Regul. 71, 295–299. doi: 10.1007/s10725-013-9828-1

[B15] Ding Y. Q. Fan K. Wang Y. Fang W. P. Zhu X. J. Chen L.. (2022). Drought and heat stress-mediated modulation of alternative splicing in the genes involved in biosynthesis of metabolites related to tea quality. Mol. Biol. 56, 257–268. doi: 10.1134/S0026893322020042 35403623

[B16] Du Y. Y. Chen H. Zhong W. L. Wu L. Y. Ye J. H. Lin C.. (2008). Effect of temperature on accumulation of chlorophylls and leaf ultrastructure of low temperature induced albino tea plant. Afr. J. Biotechnol. 7, 1881–1885. doi: 10.5897/AJB2008.000-5036

[B17] Du Y. Y. Liang Y. R. Wang H. Wang K. R. Lu J. L. Zhang G. H.. (2006). A study on the chemical composition of albino tea cultivars. J. Hortic. Sci. Biotechnol. 81, 809–812. doi: 10.1080/14620316.2006.11512142

[B18] Egghe L. (2006). Theory and practise of the g-index. Scientometrics 69, 131–152. doi: 10.1007/s11192-006-0144-7

[B19] Eungwanichayapant P. D. Popluechai S. (2009). Accumulation of catechins in tea in relation to accumulation of mRNA from genes involved in catechin biosynthesis. Plant Physiol. Biochem. 47, 94–97. doi: 10.1016/j.plaphy.2008.11.002 19081728

[B20] Fan K. Fan D. Ding Z. Su Y. Wang X. (2015). Cs-miR156 is involved in the nitrogen form regulation of catechins accumulation in tea plant (Camellia sinensis l.). Plant Physiol. Biochem. 97, 350–360. doi: 10.1016/j.plaphy.2015.10.026 26520678

[B21] Feng L. Gao M. J. Hou R. Y. Hu X. Y. Zhang L. Wan X. C.. (2014). Determination of quality constituents in the young leaves of albino tea cultivars. Food Chem. 155, 98–104. doi: 10.1016/j.foodchem.2014.01.044 24594160

[B22] Fu X. Chen Y. Mei X. Katsuno T. Kobayashi E. Dong F.. (2015). Regulation of formation of volatile compounds of tea (Camellia sinensis) leaves by single light wavelength. Sci. Rep. 5, 16858. doi: 10.1038/srep16858 26567525 PMC4645219

[B23] Gao X. Zhang C. Lu C. Wang M. Xie N. Chen J.. (2021). Disruption of photomorphogenesis leads to abnormal chloroplast development and leaf variegation in camellia sinensis. Front. Plant Sci. 12. doi: 10.3389/fpls.2021.720800 PMC8459013 34567034

[B24] Guo X. Ho C. Schwab W. Song C. Wan X. (2019). Aroma compositions of large-leaf yellow tea and potential effect of theanine on volatile formation in tea. Food Chem. 280, 73–82. doi: 10.1016/j.foodchem.2018.12.066 30642509

[B25] Guo X. Song C. Ho C.-T. Wan X. (2018). Contribution of l-theanine to the formation of 2,5-dimethylpyrazine, a key roasted peanutty flavor in oolong tea during manufacturing processes. Food Chem. 263, 18–28. doi: 10.1016/j.foodchem.2018.04.117 29784304

[B26] Han Y. Huang K. Liu Y. Jiao T. Ma G. Qian Y.. (2017). Functional analysis of two flavanone-3-Hydroxylase genes from camellia sinensis: A critical role in flavonoid accumulation. GENES 8, 300. doi: 10.3390/genes8110300 29088063 PMC5704213

[B27] Hao X. Horvath D. P. Chao W. S. Yang Y. Wang X. Xiao B. (2014). Identification and evaluation of reliable reference genes for quantitative real-time PCR analysis in tea plant (Camellia sinensis (L.) o. kuntze). Int. J. Mol. Sci. 15, 22155–22172. doi: 10.3390/ijms151222155 25474086 PMC4284700

[B28] He X. Zhao X. Gao L. Shi X. Dai X. Liu Y.. (2018). Isolation and characterization of key genes that promote flavonoid accumulation in purple-leaf tea (Camellia sinensis l.). Sci. Rep. 8, 130. doi: 10.1038/s41598-017-18133-z 29317677 PMC5760735

[B29] Hidese S. Ogawa S. Ota M. Ishida I. Yasukawa Z. Ozeki M.. (2019). Effects of l-theanine administration on stress-related symptoms and cognitive functions in healthy adults: A randomized controlled trial. Nutrients 11, 2362. doi: 10.3390/nu11102362 31623400 PMC6836118

[B30] Hirsch J. E. (2005). An index to quantify an individual’s scientific research output. Proc. Natl. Acad. Sci. U.S.A. 102, 16569–16572. doi: 10.1073/pnas.0507655102 16275915 PMC1283832

[B31] Inoue K. Hiratake J. Mizutani M. Takada M. Yamamoto M. Sakata K. (2003). Beta-glycosylamidine as a ligand for affinity chromatography tailored to the glycon substrate specificity of beta-glycosidases. Carbohydr. Res. 338, 1477–1490. doi: 10.1016/s0008-6215(03)00201-5 12829393

[B32] Jiang X. Liu Y. Li W. Zhao L. Meng F. Wang Y.. (2013). Tissue-specific, development-dependent phenolic compounds accumulation profile and gene expression pattern in tea plant camellia sinensis. PloS One 8, e62315. doi: 10.1371/journal.pone.0062315 23646127 PMC3639974

[B33] Kobayashi K. Teruya T. Suenaga K. Matsui Y. Masuda H. Kigoshi H. (2006). Isotheasaponins b-1-B-3 from camellia sinensis var. sinensis tea leaves. Phytochemistry 67, 1385–1389. doi: 10.1016/j.phytochem.2006.05.025 16808937

[B34] Ku K. M. Choi J. N. Kim J. Kim J. K. Yoo L. G. Lee S. J.. (2010). Metabolomics analysis reveals the compositional differences of shade grown tea (Camellia sinensis l.). J. Agric. Food Chem. 58, 418–426. doi: 10.1021/jf902929h 19994861

[B35] Lee L.-S. Choi J. H. Son N. Kim S.-H. Park J.-D. Jang D.-J.. (2013). Metabolomic analysis of the effect of shade treatment on the nutritional and sensory qualities of green tea. J. Agric. Food Chem. 61, 332–338. doi: 10.1021/jf304161y 23256790

[B36] Li P. Fu J. Xu Y. Shen Y. Zhang Y. Ye Z.. (2022a). CsMYB1 integrates the regulation of trichome development and catechins biosynthesis in tea plant domestication. New Phytol. 234, 902–917. doi: 10.1111/nph.18026 35167117 PMC9311817

[B37] Li P. Xia E. Fu J. Xu Y. Zhao X. Tong W.. (2022b). Diverse roles of MYB transcription factors in regulating secondary metabolite biosynthesis, shoot development, and stress responses in tea plants (Camellia sinensis). Plant J 110, 1144–1165. doi: 10.1111/tpj.15729 35277905

[B38] Li C.-F. Yao M.-Z. Ma C.-L. Ma J.-Q. Jin J.-Q. Chen L. (2015a). Differential metabolic profiles during the albescent stages of “Anji baicha” (Camellia sinensis). PloS One 10, e0139996. doi: 10.1371/journal.pone.0139996 26444680 PMC4622044

[B39] Li C.-F. Zhu Y. Yu Y. Zhao Q.-Y. Wang S.-J. Wang X.-C.. (2015b). Global transcriptome and gene regulation network for secondary metabolite biosynthesis of tea plant (Camellia sinensis). BMC Genomics 16, 560. doi: 10.1186/s12864-015-1773-0 26220550 PMC4518527

[B40] Liao Y. Zhou X. Zeng L. (2022). How does tea ( *Camellia sinensis* ) produce specialized metabolites which determine its unique quality and function: A review. Crit. Rev. Food Sci. Nutr. 62, 3751–3767. doi: 10.1080/10408398.2020.1868970 33401945

[B41] Lin S. Chen Z. Chen T. Deng W. Wan X. Zhang Z. (2022). Theanine metabolism and transport in tea plants (Camellia sinensis l.): Advances and perspectives. Crit. Rev. Biotechnol. 1–15. doi: 10.1080/07388551.2022.2036692 35430936

[B42] Liu D. Mei J. Wang J. Tang R. Chen L. Ma C. (2020). Research progress on albino trait of tea plant. China Tea 42, 24–35.

[B43] Liu Y. Pang D. Jiang H. Chen C. Sun Y. Tian Y.. (2022). Identifying key genes involved in yellow leaf variation in “Menghai huangye” based on biochemical and transcriptomic analysis. Funct. Integr. Genomics 22, 251–260. doi: 10.1007/s10142-022-00829-9 35211836

[B44] Lu L. Chen H. Wang X. Zhao Y. Yao X. Xiong B.. (2021). Genome-level diversification of eight ancient tea populations in the guizhou and yunnan regions identifies candidate genes for core agronomic traits. Hortic. Res.-England 8, 190. doi: 10.1038/s41438-021-00617-9 PMC8355299 34376642

[B45] Lv Z. Zhang C. Shao C. Liu B. Liu E. Yuan D.. (2021). Research progress on the response of tea catechins to drought stress. J. Sci. Food Agric. 101, 5305–5313. doi: 10.1002/jsfa.11330 34031895

[B46] Ma S. J. Watanabe N. Yagi A. Sakata K. (2001). The (3R,9R)-3-hydroxy-7,8-dihydro-beta-ionol disaccharide glycoside is an aroma precursor in tea leaves. Phytochemistry 56, 819–825. doi: 10.1016/s0031-9422(00)00361-7 11324911

[B47] Mamati G. E. Liang Y. Lu J. (2006). Expression of basic genes involved in tea polyphenol synthesis in relation to accumulation of catechins and total tea polyphenols. J. Sci. Food Agric. 86, 459–464. doi: 10.1002/jsfa.2368

[B48] Mei Y. Liang X. (2022). World tea production and sales situation report. Available at: https://www.ctma.com.cn/xiehuidongtai/72288.html (Accessed 29, 2022).

[B49] Mei X. Liu X. Zhou Y. Wang X. Zeng L. Fu X.. (2017). Formation and emission of linalool in tea (Camellia sinensis) leaves infested by tea green leafhopper (Empoasca (Matsumurasca) onukii matsuda). Food Chem. 237, 356–363. doi: 10.1016/j.foodchem.2017.05.124 28764007

[B50] Morikawa H. Okuda K. Kunihira Y. Inada A. Miyagi C. Matsuo Y.. (2019). Oligomerization mechanism of tea catechins during tea roasting. Food Chem. 285, 252–259. doi: 10.1016/j.foodchem.2019.01.163 30797342

[B51] Naranan S. (1970). Bradford’s law of bibliography of science: an interpretation. Nature 227, 631–632. doi: 10.1038/227631a0 5429302

[B52] Pao M. L. (1985). Lotka’s law: A testing procedure. Inf. Process. Manage. 21, 305–320. doi: 10.1016/0306-4573(85)90055-X

[B53] Pei Z. Chen S. Ding L. Liu J. Cui X. Li F.. (2022). Current perspectives and trend of nanomedicine in cancer: A review and bibliometric analysis. J. Control Release 352, 211–241. doi: 10.1016/j.jconrel.2022.10.023 36270513

[B54] Provart N. J. Alonso J. Assmann S. M. Bergmann D. Brady S. M. Brkljacic J.. (2016). 50 years of arabidopsis research: highlights and future directions. New Phytol. 209, 921–944. doi: 10.1111/nph.13687 26465351

[B55] Qiu J. Zhao R. Yang S. Dong K. (2017). Author distribution of literature information: Lotka’s law. Informetrics, 145–183. doi: 10.1007/978-981-10-4032-0_6

[B56] Rubel Mozumder N. H. M. Hwang K. H. Lee M.-S. Kim E.-H. Hong Y.-S. (2021). Metabolomic understanding of the difference between unpruning and pruning cultivation of tea (Camellia sinensis) plants. Food Res. Int. 140, 109978. doi: 10.1016/j.foodres.2020.109978 33648213

[B57] Shao C. Deng Z. Liu J. Li Y. Zhang C. Yao S.. (2022). Effects of preharvest shading on dynamic changes in metabolites, gene expression, and enzyme activity of three tea types during processing. J. Agric. Food Chem. 70, 14544–14558. doi: 10.1021/acs.jafc.2c05456 36321848

[B58] Shao C. Zhang C. Lv Z. Shen C. (2021). Pre-and post-harvest exposure to stress influence quality-related metabolites in fresh tea leaves (Camellia sinensis). Scientia Hortic. 281, 109984. doi: 10.1016/j.scienta.2021.109984

[B59] Sharma E. Joshi R. Gulati A. (2018). L-theanine: An astounding sui generis integrant in tea. Food Chem. 242, 601–610. doi: 10.1016/j.foodchem.2017.09.046 29037735

[B60] Shen J. Zou Z. Zhang X. Zhou L. Wang Y. Fang W.. (2018). Metabolic analyses reveal different mechanisms of leaf color change in two purple-leaf tea plant (Camellia sinensis l.) cultivars. Hortic. Res.-England 5, 7. doi: 10.1038/s41438-017-0010-1 PMC5802758 29423237

[B61] Shi C.-Y. Yang H. Wei C.-L. Yu O. Zhang Z.-Z. Jiang C.-J.. (2011). Deep sequencing of the camellia sinensis transcriptome revealed candidate genes for major metabolic pathways of tea-specific compounds. BMC Genomics 12, 131. doi: 10.1186/1471-2164-12-131 21356090 PMC3056800

[B62] Shu W. S. Zhang Z. Q. Lan C. Y. Wong M. H. (2003). Fluoride and aluminium concentrations of tea plants and tea products from sichuan province, PR China. Chemosphere 52, 1475–1482. doi: 10.1016/S0045-6535(03)00485-5 12867178

[B63] Sun L. Zhang M. Liu X. Mao Q. Shi C. Kochian L. V.. (2020). Aluminium is essential for root growth and development of tea plants (Camellia sinensis). J. Integr. Plant Biol. 62, 984–997. doi: 10.1111/jipb.12942 32320136 PMC7383589

[B64] Venable G. T. Shepherd B. A. Loftis C. M. McClatchy S. G. Roberts M. L. Fillinger M. E.. (2016). Bradford’s law: identification of the core journals for neurosurgery and its subspecialties. J. Neurosurg. 124, 569–579. doi: 10.3171/2015.3.JNS15149 26339849

[B65] Wan Y. Shen J. Ouyang J. Dong P. Hong Y. Liang L.. (2022). Bibliometric and visual analysis of neutrophil extracellular traps from 2004 to 2022. Front. Immunol. 13. doi: 10.3389/fimmu.2022.1025861 PMC9634160 36341351

[B66] Wang X. Feng H. Chang Y. Ma C. Wang L. Hao X.. (2020). Population sequencing enhances understanding of tea plant evolution. Nat. Commun. 11, 4447. doi: 10.1038/s41467-020-18228-8 32895382 PMC7477583

[B67] Wang Y. Gao L. Shan Y. Liu Y. Tian Y. Xia T. (2012a). Influence of shade on flavonoid biosynthesis in tea (Camellia sinensis (L.) o. kuntze). Scientia Hortic. 141, 7–16. doi: 10.1016/j.scienta.2012.04.013

[B68] Wang Y. Gao L. Wang Z. Liu Y. Sun M. Yang D.. (2012b). Light-induced expression of genes involved in phenylpropanoid biosynthetic pathways in callus of tea (Camellia sinensis (L.) o. kuntze). Scientia Hortic. 133, 72–83. doi: 10.1016/j.scienta.2011.10.017

[B69] Wang Y. Kan Z. Thompson H. J. Ling T. Ho C.-T. Li D.. (2019). Impact of six typical processing methods on the chemical composition of tea leaves using a single *Camellia sinensis* cultivar, longjing 43. J. Agric. Food Chem. 67, 5423–5436. doi: 10.1021/acs.jafc.8b05140 30403138

[B70] Wang Y.-X. Liu Z.-W. Wu Z.-J. Li H. Wang W.-L. Cui X.. (2018). Genome-wide identification and expression analysis of GRAS family transcription factors in tea plant (Camellia sinensis). Sci. Rep. 8, 3949. doi: 10.1038/s41598-018-22275-z 29500448 PMC5834537

[B71] Wang W. Xin H. Wang M. Ma Q. Wang L. Kaleri N. A.. (2016). Transcriptomic analysis reveals the molecular mechanisms of drought-Stress-Induced decreases in camellia sinensis leaf quality. Front. Plant Sci. 7. doi: 10.3389/fpls.2016.00385 PMC4811933 27066035

[B72] Wang P. Yu J. Jin S. Chen S. Yue C. Wang W.. (2021). Genetic basis of high aroma and stress tolerance in the oolong tea cultivar genome. Hortic. Res. 8, 107. doi: 10.1038/s41438-021-00542-x 33931633 PMC8087695

[B73] Wang X.-C. Zhao Q.-Y. Ma C.-L. Zhang Z.-H. Cao H.-L. Kong Y.-M.. (2013). Global transcriptome profiles of camellia sinensis during cold acclimation. BMC Genomics 14, 415. doi: 10.1186/1471-2164-14-415 23799877 PMC3701547

[B74] Wei T. Liu W. Zheng Z. Chen Y. Shen M. Li C. (2022). Bibliometric analysis of research trends on 3-Monochloropropane-1,2-Diol esters in foods. J. Agric. Food Chem. 70, 15347–15359. doi: 10.1021/acs.jafc.2c06067 36468534

[B75] Wei C. Yang H. Wang S. Zhao J. Liu C. Gao L.. (2018). Draft genome sequence of camellia sinensis var. sinensis provides insights into the evolution of the tea genome and tea quality. Proc. Natl. Acad. Sci. U.S.A. 115, E4151–E4158. doi: 10.1073/pnas.1719622115 29678829 PMC5939082

[B76] Wen B. Li J. Luo Y. Zhang X. Wang K. Liu Z.. (2020). Identification and expression profiling of MYB transcription factors related to l-theanine biosynthesis in camellia sinensis. Int. J. Biol. Macromol 164, 4306–4317. doi: 10.1016/j.ijbiomac.2020.08.200 32861783

[B77] Wishart D. S. Tzur D. Knox C. Eisner R. Guo A. C. Young N.. (2007). HMDB: the human metabolome database. Nucleic Acids Res. 35, D521–D526. doi: 10.1093/nar/gkl923 17202168 PMC1899095

[B78] Wu Z.-J. Li X.-H. Liu Z.-W. Xu Z.-S. Zhuang J. (2014). *De novo* assembly and transcriptome characterization: novel insights into catechins biosynthesis in camellia sinensis. BMC Plant Biol. 14, 277. doi: 10.1186/s12870-014-0277-4 25316555 PMC4203915

[B79] Xia E.-H. Li F.-D. Tong W. Li P.-H. Wu Q. Zhao H.-J.. (2019). Tea plant information archive: a comprehensive genomics and bioinformatics platform for tea plant. Plant Biotechnol. J. 17, 1938–1953. doi: 10.1111/pbi.13111 30913342 PMC6737018

[B80] Xia E.-H. Tong W. Wu Q. Wei S. Zhao J. Zhang Z.-Z.. (2020). Tea plant genomics: achievements, challenges and perspectives. Hortic. Res.-England 7, 7. doi: 10.1038/s41438-019-0225-4 PMC6938499 31908810

[B81] Xia E.-H. Zhang H.-B. Sheng J. Li K. Zhang Q.-J. Kim C.. (2017). The tea tree genome provides insights into tea flavor and independent evolution of caffeine biosynthesis. Mol. Plant 10, 866–877. doi: 10.1016/j.molp.2017.04.002 28473262

[B82] Xie Z. M. Ye Z. H. Wong M. H. (2001). Distribution characteristics of fluoride and aluminum in soil profiles of an abandoned tea plantation and their uptake by six woody species. Environ. Int. 26, 341–346. doi: 10.1016/s0160-4120(01)00010-1 11392749

[B83] Yang Z. Baldermann S. Watanabe N. (2013). Recent studies of the volatile compounds in tea. Food Res. Int. 53, 585–599. doi: 10.1016/j.foodres.2013.02.011

[B84] Yang Z. Kobayashi E. Katsuno T. Asanuma T. Fujimori T. Ishikawa T.. (2012). Characterisation of volatile and non-volatile metabolites in etiolated leaves of tea (Camellia sinensis) plants in the dark. Food Chem. 135, 2268–2276. doi: 10.1016/j.foodchem.2012.07.066 22980801

[B85] Yang Y.-Z. Li T. Teng R.-M. Han M.-H. Zhuang J. (2021). Low temperature effects on carotenoids biosynthesis in the leaves of green and albino tea plant (Camellia sinensis (L.) o. kuntze). Sci. Hortic. 285, 110164. doi: 10.1016/j.scienta.2021.110164

[B86] Ye J.-H. Ye Y. Yin J.-F. Jin J. Liang Y.-R. Liu R.-Y.. (2022). Bitterness and astringency of tea leaves and products: Formation mechanism and reducing strategies. Trends Food Sci. Technol. 123, 130–143. doi: 10.1016/j.tifs.2022.02.031

[B87] Yu S. Li P. Zhao X. Tan M. Ahmad M. Z. Xu Y.. (2021). CsTCPs regulate shoot tip development and catechin biosynthesis in tea plant (Camellia sinensis). Hortic. Res. 8, 104. doi: 10.1038/s41438-021-00538-7 33931613 PMC8087681

[B88] Yu X.-L. Sun D.-W. He Y. (2020). Emerging techniques for determining the quality and safety of tea products: A review. Compr. Rev. Food. Sci. Food Saf. 19, 2613–2638. doi: 10.1111/1541-4337.12611 33336976

[B89] Yu Z. Yang Z. (2020). Understanding different regulatory mechanisms of proteinaceous and non-proteinaceous amino acid formation in tea ( *Camellia sinensis* ) provides new insights into the safe and effective alteration of tea flavor and function. Crit. Rev. Food Sci. Nutr. 60, 844–858. doi: 10.1080/10408398.2018.1552245 30614265

[B90] Zeng L. Watanabe N. Yang Z. (2019). Understanding the biosyntheses and stress response mechanisms of aroma compounds in tea ( *Camellia sinensis* ) to safely and effectively improve tea aroma. Crit. Rev. Food Sci. Nutr. 59, 2321–2334. doi: 10.1080/10408398.2018.1506907 30277806

[B91] Zeng L. Zhou Y. Fu X. Mei X. Cheng S. Gui J.. (2017). Does oolong tea (Camellia sinensis) made from a combination of leaf and stem smell more aromatic than leaf-only tea? contribution of the stem to oolong tea aroma. Food Chem. 237, 488–498. doi: 10.1016/j.foodchem.2017.05.137 28764024

[B92] Zhang L. Cao Q.-Q. Granato D. Xu Y.-Q. Ho C.-T. (2020d). Association between chemistry and taste of tea: A review. Trends Food Sci. Technol. 101, 139–149. doi: 10.1016/j.tifs.2020.05.015

[B93] Zhang X. Chen S. Shi L. Gong D. Zhang S. Zhao Q.. (2021). Haplotype-resolved genome assembly provides insights into evolutionary history of the tea plant camellia sinensis. Nat. Genet. 53, 1250–1259. doi: 10.1038/s41588-021-00895-y 34267370 PMC8346365

[B94] Zhang C. He Q. Wang M. Gao X. Chen J. Shen C. (2020a). Exogenous indole acetic acid alleviates cd toxicity in tea (Camellia sinensis). Ecotoxicology Environ. Saf. 190, 110090. doi: 10.1016/j.ecoenv.2019.110090 31874405

[B95] Zhang L. Ho C.-T. Zhou J. Santos J. S. Armstrong L. Granato D. (2019). Chemistry and biological activities of processed camellia sinensis teas: A comprehensive review. Compr. Rev. Food Sci. Food Saf. 18, 1474–1495. doi: 10.1111/1541-4337.12479 33336903

[B96] Zhang C. Wang M. Gao X. Zhou F. Shen C. Liu Z. (2020b). Multi-omics research in albino tea plants: Past, present, and future. Scientia Hortic. 261, 108943. doi: 10.1016/j.scienta.2019.108943

[B97] Zhang C. Wang M. Gao X. Zhou F. Shen C. Liu Z. (2020c). Multi-omics research in albino tea plants: past, present, and future. Scientia Hortic. 261, 108943. doi: 10.1016/j.scienta.2019.108943

[B98] Zheng Z. Xu W. Xu Y. Xue Q. (2022). Mapping knowledge structure and themes trends of biodegradable mg-based alloy for orthopedic application: A comprehensive bibliometric analysis. Front. Bioeng Biotechnol. 10. doi: 10.3389/fbioe.2022.940700 PMC9395602 36017343

[B99] Zhou Y. Zeng L. Liu X. Gui J. Mei X. Fu X.. (2017). Formation of (E)-nerolidol in tea (Camellia sinensis) leaves exposed to multiple stresses during tea manufacturing. Food Chem. 231, 78–86. doi: 10.1016/j.foodchem.2017.03.122 28450026

[B100] Zhou Y. Zhang Z. Bao Z. Li H. Lyu Y. Zan Y.. (2022). Graph pangenome captures missing heritability and empowers tomato breeding. Nature 606, 527–534. doi: 10.1038/s41586-022-04808-9 35676474 PMC9200638

[B101] Zou C. Li R.-Y. Chen J.-X. Wang F. Gao Y. Fu Y.-Q.. (2021). Zijuan tea- based kombucha: Physicochemical, sensorial, and antioxidant profile. Food Chem. 363, 130322. doi: 10.1016/j.foodchem.2021.130322 34147900

