# Supplementary material for: Topics and trends in fresh tea (Camellia sinensis) leaf research: A comprehensive bibliometric study
Source: Front Plant Sci. 2023 Apr 6;14:1092511. doi: 10.3389/fpls.2023.1092511 (PMC10118041; doi:10.3389/fpls.2023.1092511)
Supplement: Supplementary file 1 [file DataSheet_1.pdf]

Topics and trends in fresh tea (*Camellia sinensis*) leaf research: A comprehensive bibliometric study

YiQin Chen [1,2,3,4†], YunFei Li [1,2,3,4†], ChengWen Shen[1,2,3,4*], LiZheng Xiao[1,2,3,4*]

[1]Key Laboratory of Tea Science of Ministry of Education, College of Horticulture, Hunan Agricultural University, Changsha 410128, China

[2]National Research Center of Engineering and Technology for Utilization of Botanical Functional Ingredients, Changsha 410128, China

[3]Co-Innovation Center of Education Ministry for Utilization of Botanical Functional Ingredients, Changsha 410128, China

[4]Key Laboratory for Evaluation and Utilization of Gene Resources of Horticultural Crops, Ministry of Agriculture and Rural Affairs of China, Hunan Agricultural University, Changsha 410128, China

[†]Equal contribution and first authorship

[*] **Corresponding authors**:
Chengwen Shen, Lizheng Xiao

E-mail: scw69@163.com, lz-xiao@hunau.edu.cn

Supplementary Material

Software usage and data processing procedures

Figure S1. Core journals, according to Bradford's Law

Figure S2. Country collaboration map

Figure S3. Authors' productivity according to Lotka's Law

Figure S4. Top author's scientific production over time

Figure S5. Keywords word cloud plot

Figure S6. Reference co-citation network by the time

Table S1. Summary of Top ten scientific production countries

Table S2. Summary of Top ten scientific production institutions

Table S3. Summary of Top ten scientific production authors

Table S4. Trending topics and their trending duration generated with Bibliometrix

Table S5. Summary of the clusters identified from the reference co-citation network

Table S6. Summary of essential references

Table S7. The synonymous list of Keywords

Software usage and data processing procedures

Three bibliometric tools with their required run environment are used in this research: CiteSpace version 5.8.R3, Bibliometrix version 3.2.1 with R 4.12, and VOSviewer version 1.6.18. Besides, Microsoft Excel 2021 is used for the data process.

The filtered data set was imported into CiteSpace. Then journals' co-citation network, the collaboration networks of countries, institutions, authors, keyword co-occurrence networks, and reference co-citation network were applied. The centrality value of each item was calculated by CiteSpace, based on the networks mentioned above.

While using the CiteSpace, the *period* was set to 'JAN 2001 – DEC 2021', and *#Years Per Slice* was set to '3'. Pathfinder was selected as the Pruning algorithm for a high-quality network in generating the reference co-citation network. Pruning *sliced networks* and *Pruning the merged networks* were both marked. Other parameters remained as default.

The use of Bibliometrix followed the standard guidelines provided on its official website (https://www.bibliometrix.org/home/). The filtered data set was analysed with the shiny app (the function *biblioshiny*) included in the Bibliometrix R package. Country collaboration map, Author impacts, Bradford's Law, Lotka's Law, word cloud, and trending topics were performed by *biblioshiny*. A synonymous list of keywords was loaded while processing the Trending Topics, Table S4. While analysing the trending topics, the method mentioned by [98] was applied. The trend topics analyses provided the median year for the most popular keywords, separating the higher half from the lower half of the data sample. This means that keywords with high frequency in the mid-year range were mentioned consistently throughout the period. In contrast, keywords at the beginning and end of the range represented fading and trending topics, respectively. In addition, a trend status value was defined as follows, where S presents the value of trending status:

$$S = \frac{\mathrm{year_{mid}} - \frac{\mathrm{year_{q1}} + \mathrm{year_{q3}}}{2}}{\mathrm{year_{q3}}}$$

VOSviewer was used to explore the co-occurrence of the keywords, and the following steps were followed. The Filtered data was imported into VOSviewer, the *type of analysis* was set to 'Co-occurrence，' the *Unit of analysis* was set to 'Keywords plus', and the *counting method* was set to 'full counting', a *VOSviewer thesaurus* with the exact content of Table S4 was loaded. Further, the *Minimum number of occurrences of a keyword* was set to '10', resulting in 104 keywords meeting the threshold. Other parameters remained as default.

Figures

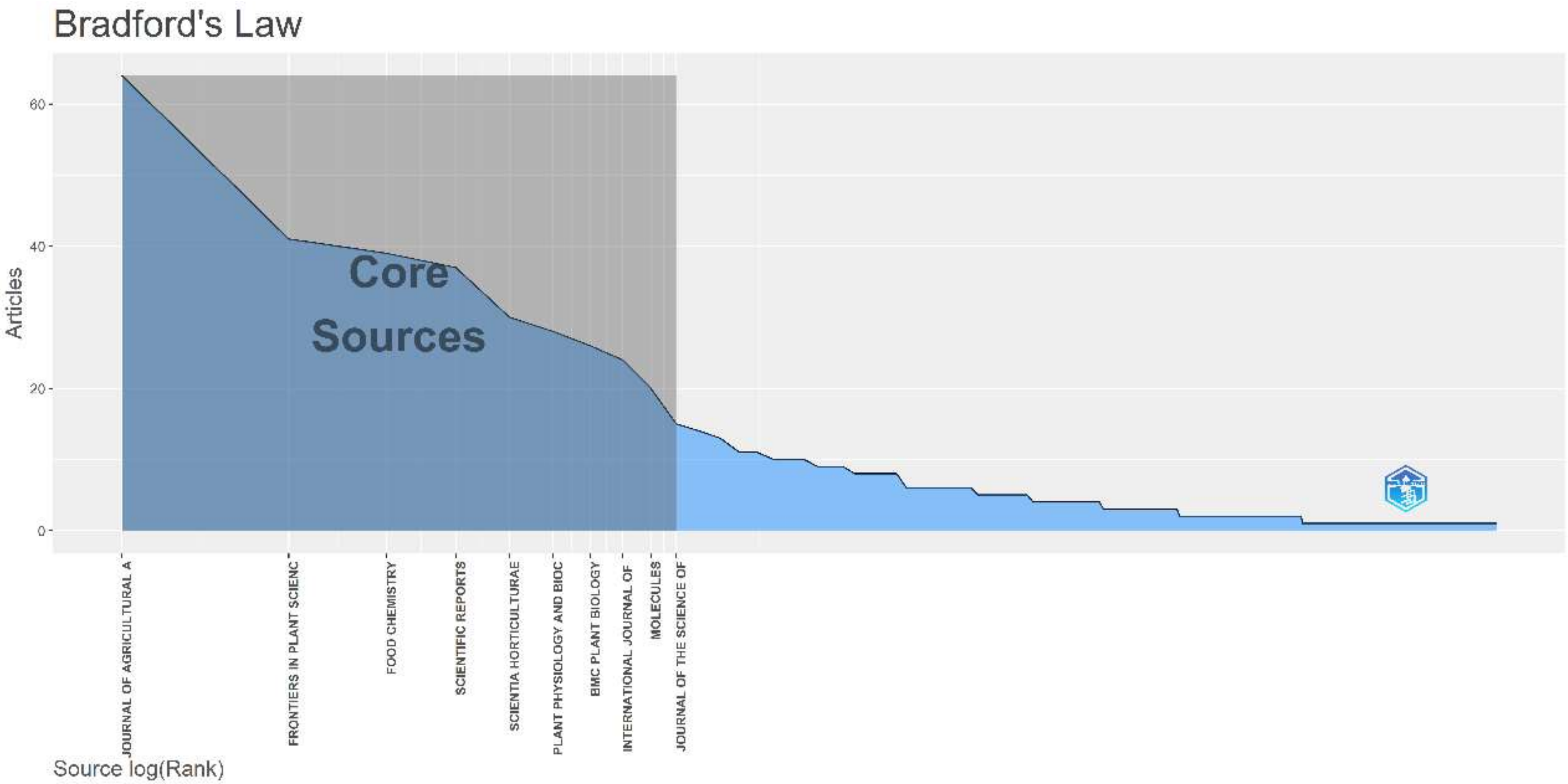

Figure S1 Core journals according to Bradford's Law, generated by Bibliometrix.

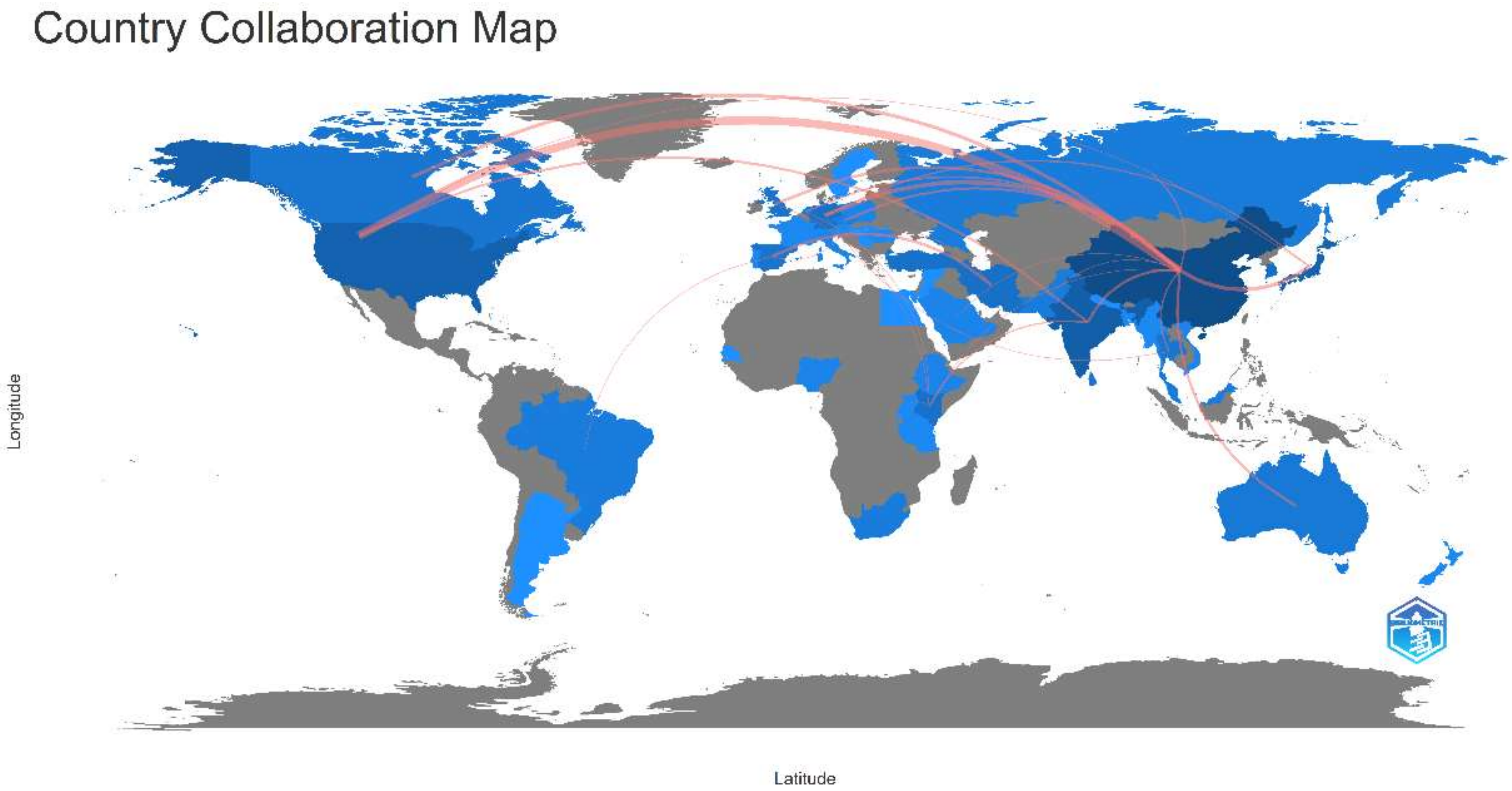

Figure S2 Country collaboration map. The countries' color on the map represents the publications' number; the darker the color, the more publications, contrary to that. And the pink connecting line represents the number of papers published in collaboration between two countries.

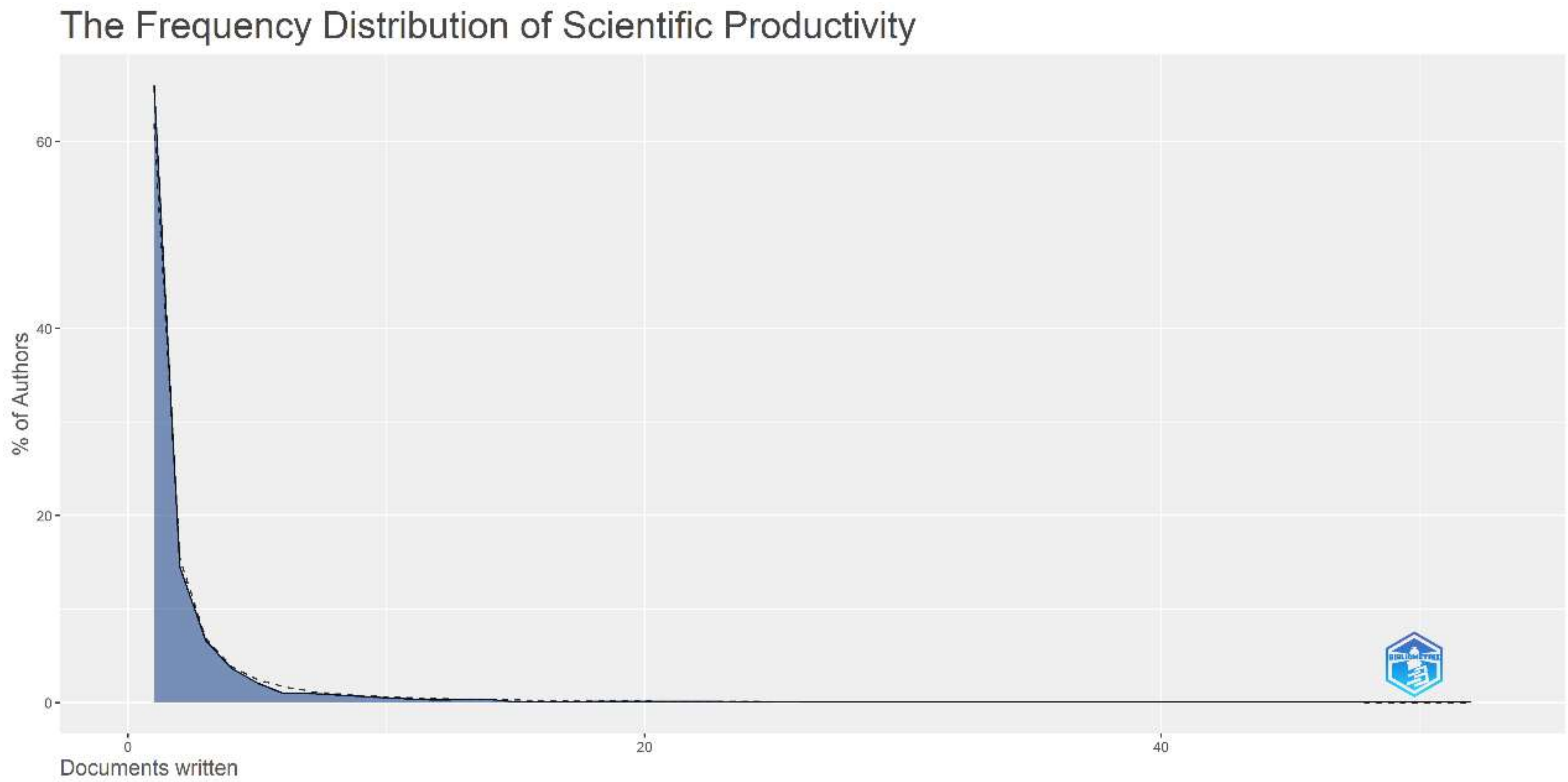

Figure S3 Author productivity according to Lotka’s Law, generated by Bibliometrix. The black dashed line indicates the predicted value of Lotka's Law, and the blue part is the author produced in the field of TFL research.

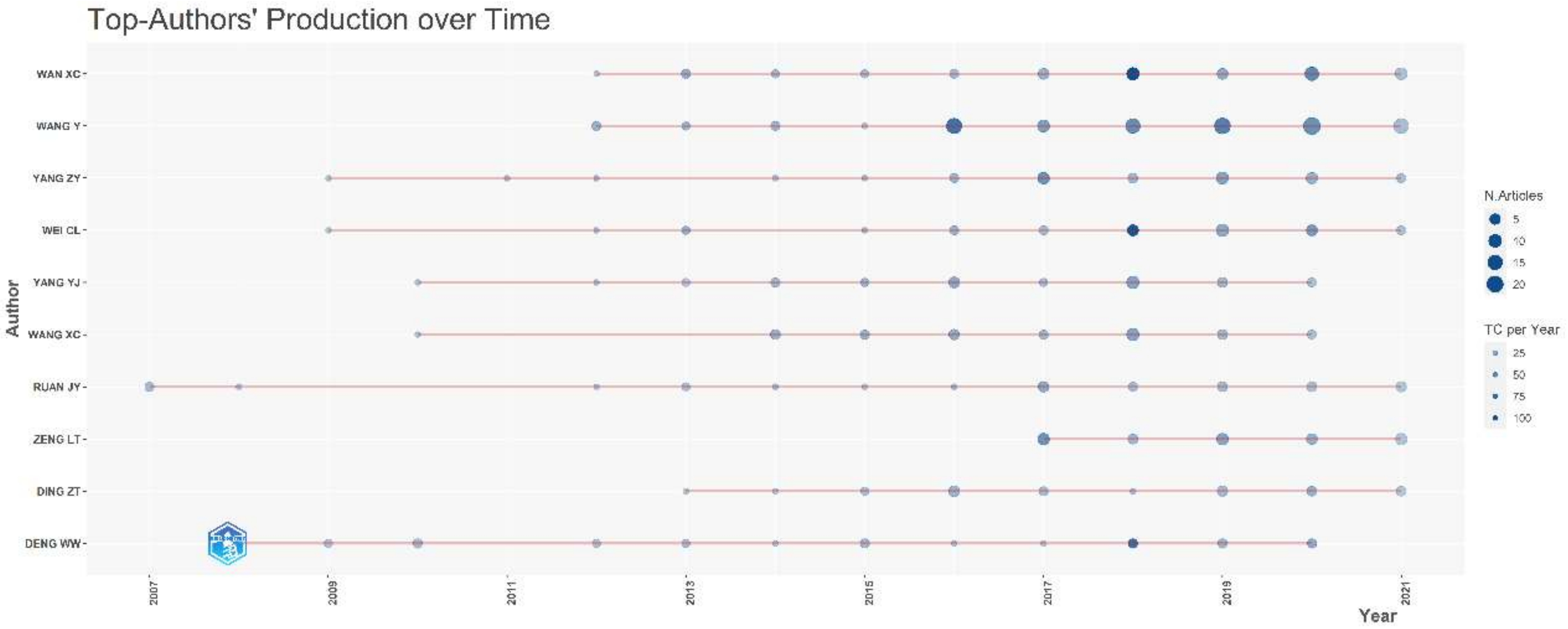

Figure S4 Top ten authors' scientific production over time, generated with Bibliometrix. The size and color of the bubbles present the number of publications and total citations during the year.

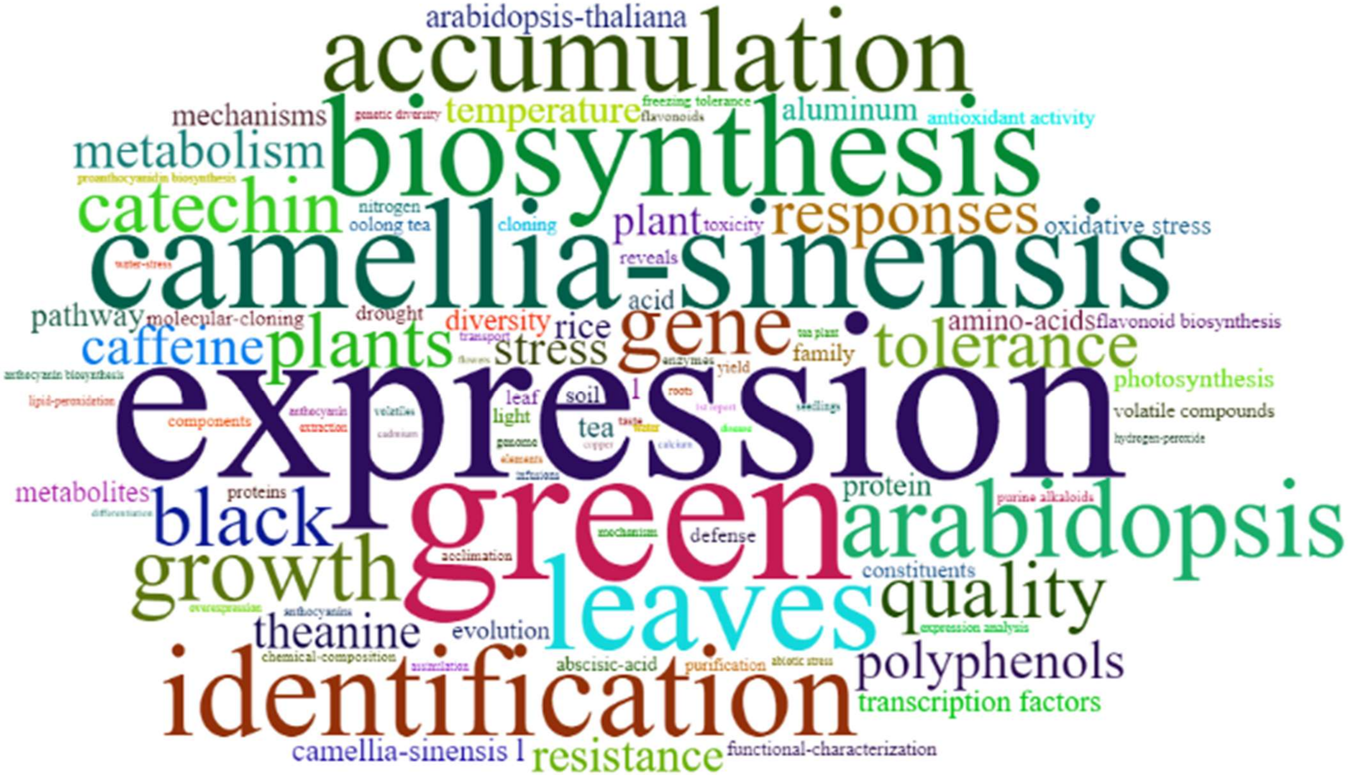

Figure S5 Word cloud plot of keywords generated by Bibliometrix; the size of keywords indicates their frequency of occurrence.

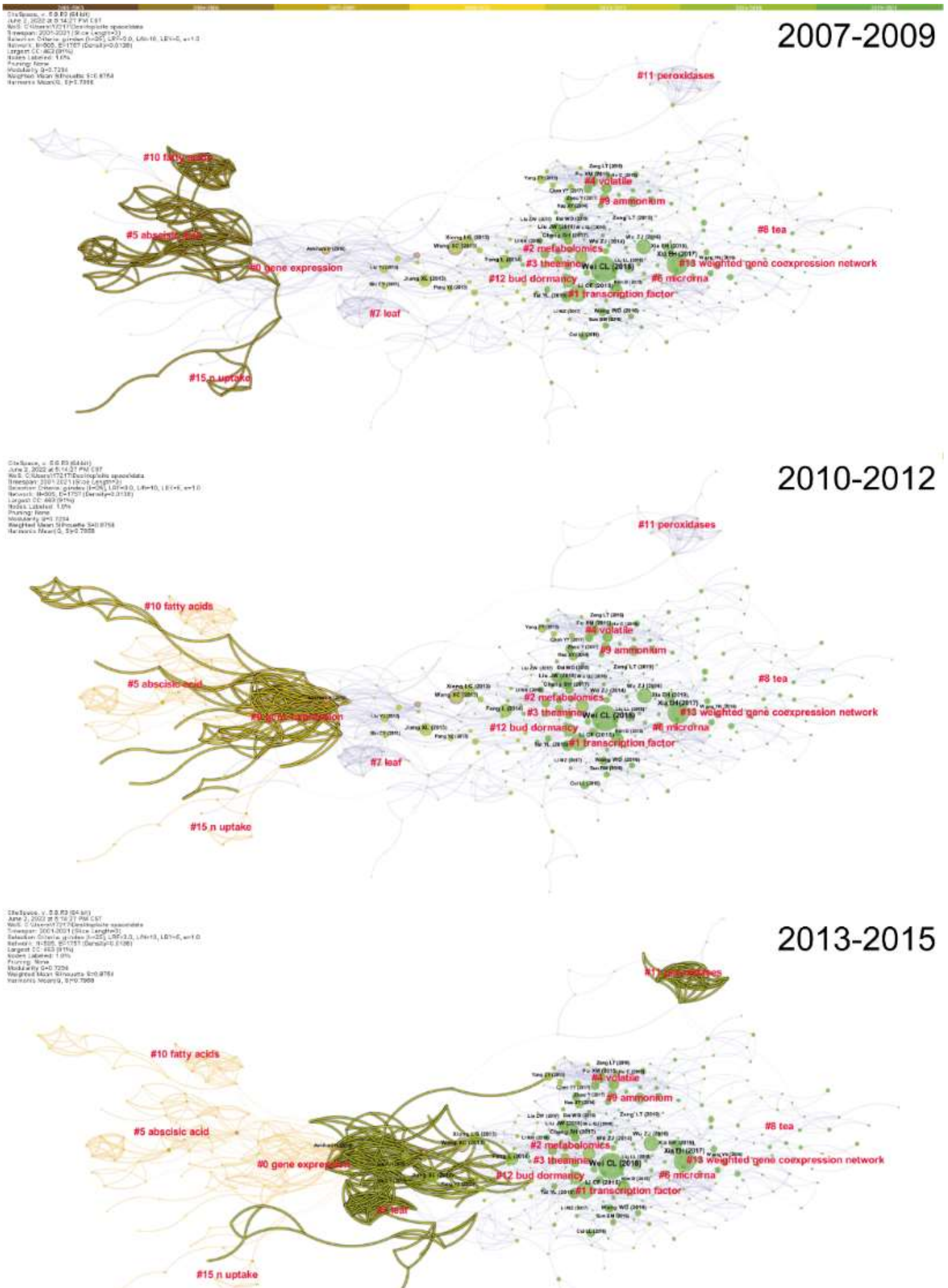
2007-2009
#11 peroxidases
#10 fatty acids
#5 abscisic acid
#0 gene expression
#7 leaf
#15 n uptake
#4 volatile
#9 ammonium
#2 metabolomics
#3 theanine
Wei CL (2018)
#12 bud dormancy
#1 transcription factor
#6 microrna
#13 weighted gene coexpression network
#8 tea
2010-2012
2013-2015

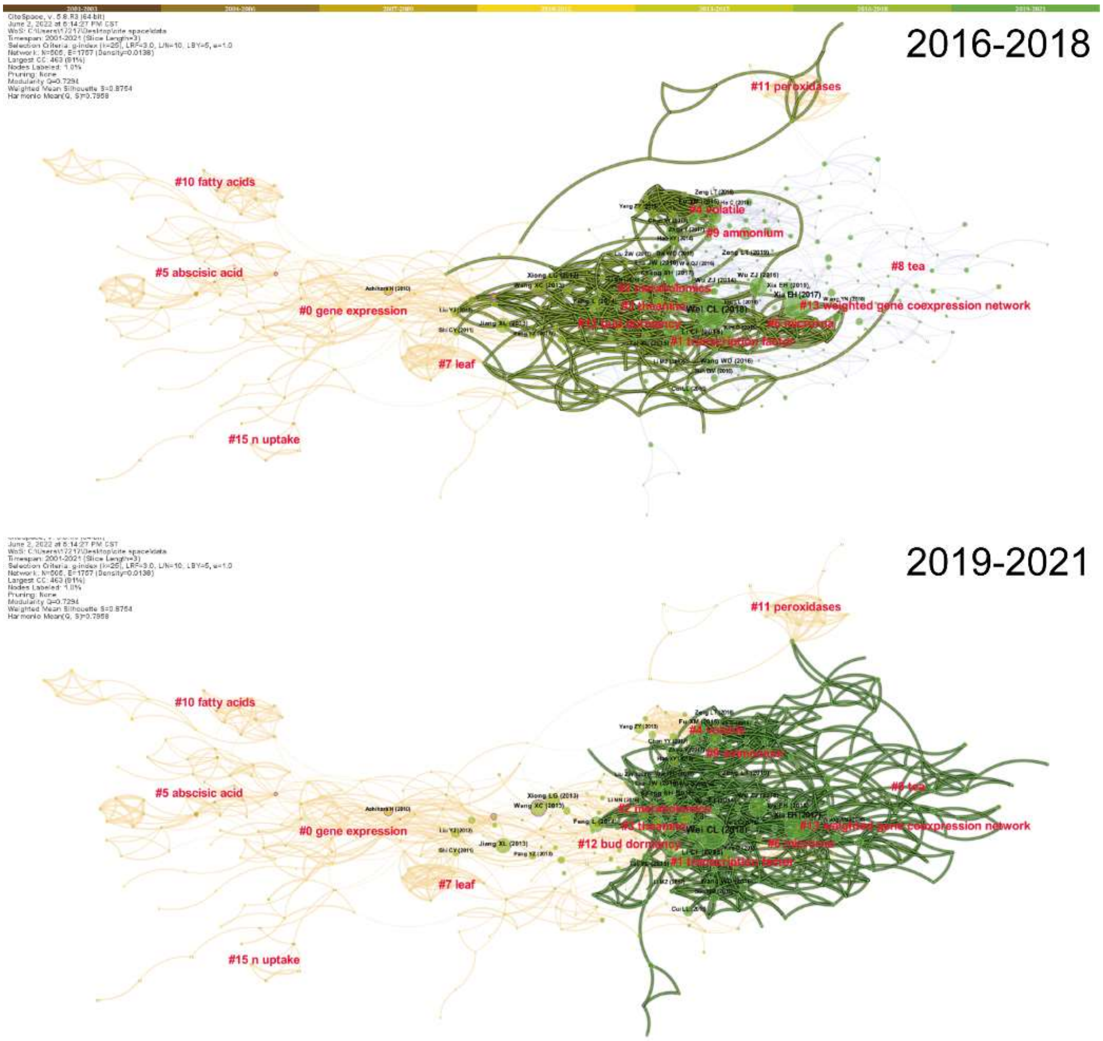

Figure S6 Reference co-citation networks over time. Generated with CiteSpace, the time slice was set to 3 years, and no pruning algorithm was selected.

Tables

Table S1 Top ten countries by the number of publications.

Country/regions	NP	SCP	MCP	MCP (%)	TGC	Centrality
1. China	638	522	116	18.18%	9748	0.97
2. India	103	94	9	8.74%	1291	0.28
3. Japan	59	48	11	18.64%	1194	0.17
4. South Korea	22	18	4	18.18%	288	0.04
5. Turkey	17	16	1	5.88%	153	0.00
6. USA	17	6	11	64.71%	234	0.06
7. Iran	15	12	3	20.00%	101	0.01
8. Sri Lanka	11	11	0	0.00%	72	0.01
9. Germany	7	0	7	100.00%	180	0.12
10. Kenya	7	6	1	14.29%	117	0.18

NP, SCP, MCP, MCP percentage, and TGC represent the number of publications, the number of co-authored papers by authors of the same nationality, the number of co-authored pieces with authors from other countries, the rate of international cooperation, total global citations, respectively.

Table S2 Top ten institutions by the number of publications.

Institution	Country/region	NP	TGC	h-index	g-index	Centrality	PY-start
1. Chinese Academy of Agricultural Sciences	China	205	3762	33	45	0.25	2007
2. Anhui Agricultural University	China	192	3855	30	52	0.20	2008
3. Fujian Agricultural and Forestry University	China	89	1521	21	33	0.07	2009
4. Chinese Academy of Sciences	China	86	1600	21	35	0.36	2009
5. Nanjing Agricultural University	China	76	1529	23	36	0.04	2014
6. Zhejiang University	China	55	963	19	29	0.17	2007
7. Huazhong Agricultural University	China	40	475	12	19	0.03	2007
8. Qingdao Agricultural University	China	36	514	15	21	0.10	2013
9. University of Chinese Academy of Sciences	China	34	734	16	26	0.06	2015
10.Hunan Agricultural University	China	27	406	13	23	0.06	2013

NP, TGC, and PY represent the number of publications, total global citations, and publication year.

Table S3 Top ten authors by the number of publications.

Authors	Working institutions	NP	TGC	TLC	h-index	g-index	Centrality	PY-start
1. Xiaochun Wan	Anhui Agricultural University	52	1300	446	18	35	0.11	2012
2. Yu Wang	Qingdao Agricultural University	47	435	123	13	19	0.08	-
3. Ziyin Yang	South China Botanical Garden, Chinese Academy of Sciences	37	858	268	16	28	0.07	2009
4. Chaoling Wei	Anhui Agricultural University	35	1143	390	18	33	0.23	2009
5. Yajun Yang	Tea Research Institute, Chinese Academy of Agricultural Sciences	33	680	202	16	25	0.00	2010
6. Xinchao Wang	Tea Research Institute, Chinese Academy of Agricultural Sciences	32	709	205	16	26	0.09	2010
7. Jianyun Ruan	Tea Research Institute, Chinese Academy of Agricultural Sciences	31	651	209	14	25	0.01	2007
8. Lanting Zeng	South China Botanical Garden, Chinese Academy of Sciences	31	472	145	13	26	0.04	2017
9. Zhaotang Ding	Qingdao Agricultural University	28	314	95	12	24	0.29	2013
10. Weiwei Deng	Anhui Agricultural University	26	972	386	14	25	0.03	2008

NP, TGC, TLC, and PY represent the number of publications, total global citations, total local citations, and publication year. CiteSpace calculates centrality in the journal co-citation network.

Table S4 Trending topics and their trending duration generated with Bibliometrix, using Keywords Plus as the term.

Keywords	Frequency	Year_q1	Year_med	Year_q3
dry-matter production	5	2008	2009	2012
spectrometry	7	2008	2010	2014
multielement analysis	5	2009	2010	2020
gallate	7	2008	2011	2014
bioavailability	5	2010	2011	2017
superoxide-dismutase	8	2010	2012	2016
induction	6	2012	2012	2014
hydrogen-peroxide	11	2012	2014	2018
cadmium	10	2011	2014	2018
capacity	8	2010	2014	2020
biochemical markers	7	2012	2014	2016
cancer	7	2010	2014	2018
purine alkaloids	14	2010	2015	2019
chemical-composition	13	2010	2015	2017
chlorophyll fluorescence	9	2013	2015	2018
degradation	9	2013	2015	2017
localization	9	2011	2015	2019
acid	26	2013	2016	2019
black	23	2011	2016	2018
soil	22	2012	2016	2020
drought	20	2013	2016	2018
enzymes	14	2012	2016	2020
catechin	74	2013	2017	2019

Keywords	Frequency	Year_q1	Year_med	Year_q3
caffeine	53	2015	2017	2020
polyphenols	51	2014	2017	2019
metabolism	50	2014	2017	2020
plant	41	2013	2017	2020
expression	219	2016	2018	2020
green	189	2015	2018	2020
camellia-sinensis	160	2014	2018	2020
leaf	146	2014	2018	2020
accumulation	112	2016	2018	2020
biosynthesis	135	2017	2019	2020
identification	121	2017	2019	2020
gene	87	2016	2019	2020
resistance	45	2016	2019	2020
temperature	38	2016	2019	2020
metabolites	27	2018	2020	2021
evolution	24	2018	2020	2020
expression analysis	13	2018	2020	2020
genome	12	2018	2020	2021
anthocyanin	11	2014	2020	2020

Year_q1 and Year_q2 present the start and terminate year of the keyword's trending duration.

Table S5 Summary of the clusters identified from the reference co-citation network.

Cluster-ID	Size	Label (Log-likelihood ratio algorithm; p level, LLR)	Silhouette score	Mean (Year)
#0	69	gene expression (13.78, 0.001); catechins (13.13, 0.001); theaceae (10.1, 0.005); tea plant (10.08, 0.005); anthocyanidin reductase (9.59, 0.005)	0.787	2009
#1	59	transcription factor (12.12, 0.001); flavonoid biosynthesis (11.47, 0.001); catechin biosynthesis (9.85, 0.005); anthocyanins (8, 0.005); aroma (7.74, 0.01)	0.747	2016
#2	47	metabolomics (12.24, 0.001); chlorophyll deficiency (8.63, 0.005); chlorophylls (8.58, 0.005); chlorotic mutation (8.58, 0.005); nmr (8.58, 0.005)	0.868	2015
#3	42	theanine (12.82, 0.001); brassinosteroids (8.69, 0.005); amino acid transporter (8.69, 0.005); phenylpropanoid pathway (8.69, 0.005); transport (8.69, 0.005)	0.842	2016
#4	38	volatile (71.5, 1.0E-4); aroma (68.54, 1.0E-4); tea (33.06, 1.0E-4); linalool (13.23, 0.001); signaling (13.23, 0.001)	0.924	2016
#5	32	abscisic acid (8.32, 0.005); differential display (8.32, 0.005); etioplast (8.32, 0.005); colour difference (8.32, 0.005); race (8.32, 0.005)	0.981	2005
#6	29	microrna (16.57, 1.0E-4); reactive oxygen species (11.03, 0.001); systemic root response (11.03, 0.001); leaf herbivory (11.03, 0.001); ectropis oblique (11.03, 0.001)	0.888	2014
#7	26	leaf (8.23, 0.005); antioxidant activity (7.83, 0.01); ssr marker (7.83, 0.01); est-ssr (7.83, 0.01); anthocyanin accumulation (7.83, 0.01)	0.953	2011
#8	25	tea (6.72, 0.01); tea plant (5.77, 0.05); colletotrichum camelliae (5.6, 0.05); transcription factors (tfs) (5.6, 0.05); histone deacetylases (hdacs) (4.6, 0.05)	0.896	2017
#9	21	ammonium (12.56, 0.001); fluoride (10.83, 0.005); glutamine synthetase (9.67, 0.005); tea leaves (8.83, 0.005); pectin (6.27, 0.05)	0.929	2017
#10	18	fatty acids (18.71, 1.0E-4); location of production (18.71, 1.0E-4); plucking interval (9.3, 0.005); soil water content (9.3, 0.005); nitrogenous fertilizer application rates (9.3, 0.005)	0.989	2006

Cluster-ID	Size	Label (Log-likelihood ratio algorithm; p level, LLR)	Silhouette score	Mean (Year
#11	17	peroxidases (14.73, 0.001); aluminum (10.96, 0.001); camellia sinensis (9.11, 0.005); superoxide dismutase (7.34, 0.01); peroxidase (7.34, 0.01)	0.991	2012
#12	16	bud dormancy (16.47, 1.0E-4); tea plant (10.52, 0.005); tea plant (camellia sinensis) (7.83, 0.01); gene expression (6.93, 0.01); tea (6.2, 0.05)	0.915	2014
#13	15	weighted gene co-expression network (wgcna) (6.99, 0.01); correlation (6.99, 0.01); feeding (6.99, 0.01); purine alkaloids (6.99, 0.01); laccase gene family (6.99, 0.01)	0.916	2019
#15	9	n uptake (9.3, 0.005); clone (9.3, 0.005); organic matter (9.3, 0.005); seedlings (9.3, 0.005); micronutrients (9.3, 0.005)	0.983	2005

Table S6 Summary of essential references based on Centrality in reference co-citation network, identified with CiteSpace.

Title	Author Year	Keywords	Source	Cluster-ID	Centrality
Expression of basic genes involved in tea polyphenol synthesis in relation to accumulation of catechins and total tea polyphenols	Mamati GE, 2006	Polyphenols; catechins; RT-PCR; HPLC	Journal Science Food Agriculture	#5	0.1
Accumulation of catechins in tea in relation to accumulation of mRNA from genes involved in catechin biosynthesis	Eungwanicha-yapant PD, 2009	Catechins; RT-PCR; HPLC	Plant Physiology and Biochemistry	#0	0.2
Distribution and biosynthesis of flavan-3-ols in *Camellia sinensis* seedlings and expression of genes encoding biosynthetic enzymes	Ashihara H, 2010	Flavan-3-ols; Distribution; Biosynthesis	Phytochemistry	#0	0.1
Light-induced expression of genes involved in phenylpropanoid biosynthetic pathways in callus of tea (*Camellia sinensis* (L.) O. Kuntze)	Wang YS, 2012	Light inducement; phenylpropanoid biosynthesis; gene expression	Scientia Horticulturae	#0	0.18
Effect of shade treatment on theanine biosynthesis in *Camellia sinensis* seedlings	Deng WW, 2013	Theanine synthetase; shade; amino acid	Plant Growth Regulation	#0	0.16
Global transcriptome profiles of *Camellia sinensis* during cold acclimation	Wang XC, 2013	Cold acclimation; RNA-Seq; DGE;	BMC Genomics	#12	0.11

Identification and evaluation of reliable reference genes for quantitative real-time PCR analysis in tea plant (*Camellia sinensis* (L.) O. Kuntze)	Hao XY, 2014	Reference gene; qRT-PCR; gene expression	International Journal of Molecular Sciences	#4	0.1
Cs-miR156 is involved in the nitrogen form regulation of catechins accumulation in tea plant (*Camellia sinensis* L.)	Fan K, 2015	Nitrogen forms; Catechins; Post-transcriptional regulation	Plant Physiology and Biochemistry	#9	0.1

Table S7 the synonymous list of keywords used in trending topics identification and keyword co-occurrence network generation.

Keywords	Replaced by
black teas	black
green tea	green
leaves	leaf
oolong tea	oolong
genes	gene
catechins	catechin
l-theanine	theanine
camellia-sinensis l	camellia-sinensis
camellia-sinensis l.	camellia-sinensis
plant camellia-sinensis	camellia-sinensis
l.o.-kuntze	camellia-sinensis
l. o.-kuntze	camellia-sinensis
o.kuntze	camellia-sinensis
plant camellia-sinensis	camellia-sinensis
arabidopsis-thaliana	arabidopsis
gene-expression	expression
volatile compunds	volatile
